# Syntheses, Single-Crystal Structures, and Structural Chemistry of Hexafluoridouranates(V), *M*UF_6_ (*M* = Li–Cs, Ag, Tl, H_3_O), and the Dodecafluoridodiuranate(V) Ba[U_2_F_12_]·1.36HF

**DOI:** 10.1021/acs.inorgchem.5c03005

**Published:** 2025-09-04

**Authors:** Benjamin Scheibe, Tobias B. Wassermann, Florian Kraus

**Affiliations:** Arbeitsgruppe Fluorchemie, Anorganische Chemie, Fachbereich Chemie, 9377 Philipps-Universität Marburg, Hans-Meerwein-Str. 4, 35032 Marburg, Germany

## Abstract

We present the syntheses of the hexafluoridouranates­(V) *M*UF_6_ (*M* = Li–Cs, Ag, Tl, H_3_O) and of the dodecafluoridodiuranate­(V) Ba­[U_2_F_12_]·1.36HF. With the exception of AgUF_6_ and H_3_OUF_6_, all compounds were synthesized by reacting the respective metal fluorides with β-UF_5_ in anhydrous hydrogen fluoride (aHF). AgUF_6_ was obtained as a side product in the oxidation of Ag powder with UF_6_ under a CO atmosphere, while H_3_OUF_6_ was obtained from the controlled hydrolysis of β-UF_5_ with SiO_2_ in aHF. For this hydrolysis, silica glass wool proved to be the superior choice of SiO_2_. X-ray diffraction experiments on single crystals of the compounds as well as on polycrystalline samples allowed the unambiguous determination of their crystal structures, clarifying previously published structure models that were based on only powder X-ray diffraction. The structural chemistry of these compounds is discussed. In the case of LiUF_6_, NaUF_6_, and H_3_OUF_6_, molecular UF_6_^–^ anions are observed in the solid state, while one-dimensional infinite strands of _∞_^1^{[UF_4/1_F_4/2_]^−^} anions are present in the crystal structures of KUF_6_, RbUF_6_, TlUF_6_, and AgUF_6_. The dodecafluoridodiuranate­(V) Ba­[U_2_F_12_]·1.36HF is the second example of a compound containing the peculiar [U_2_F_12_]^2–^ anion. Its U atoms show a coordination number of seven in a capped trigonal prismatic coordination sphere, and these prisms share a common edge. In contrast to the Sr homologue, the Ba compound contains HF molecules of crystallization.

## Graphical abstract

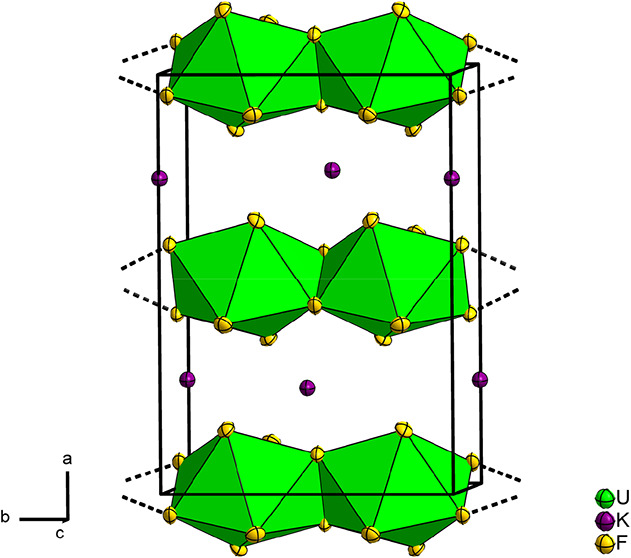

## Introduction

Two allotropic forms of uranium­(V) fluoride are known, a low-temperature form, β-UF_5_, and a high-temperature form, α-UF_5_.^1,2^ β-Uranium­(V) fluoride crystallizes in the tetragonal crystal system with a network structure. The uranium atoms are coordinated by eight fluorine atoms in the form of a distorted trigonal dodecahedron, and the crystal structure can be described by using the Niggli formula _∞_^3^[UF_2/1_F_6/2_]. α-UF_5_ also crystallizes in the tetragonal crystal system but with a one-dimensional infinite chain motif in which the U atoms are coordinated by six F atoms in an octahedron-like manner. The structure can be described using the Niggli formula _∞_^1^[UF_4/1_F_2/2_]. The chemical behavior of UF_5_ is related to that of Lewis acids PF_5_, AsF_5_, and SbF_5_. For example, hexa-, hepta- or octafluoridouranates­(V) are formed with fluoride ion donors such as the alkali metal fluorides.^3,4^

To the best of our knowledge, the first preparations of hexafluoridouranates­(V) of the composition *M*UF_6_ (with *M* as a monovalent cation) took place in solid-state syntheses of uranium­(V) fluoride and alkali metal fluorides, see reaction eq 1.^5^ Other methods are based on the reaction of UF_5_ with fluorides in various solvents; 48% hydrofluoric acid, anhydrous hydrogen fluoride (aHF), and acetonitrile have been described as suitable solvents.^3,4,6,7^ Both UF_5_ and the alkali metal fluorides are soluble in 48% hydrofluoric acid and acetonitrile. In contrast, only the alkali metal fluorides dissolve noticeably in aHF.

$$\begin{array}{l} {\mathrm{UF}}_{5\left(s\right)}+MF\left(s\right)\underset{\Delta T}{\to }M{\mathrm{UF}}_{6\left(s\right)}\\ M=\mathrm{Li}-\mathrm{Cs} \end{array}$$ 1

Another synthesis approach is the reduction of uranium­(VI) fluoride with one-electron donors such as nitrogen monoxide or nitrogen dioxide.^8–13^ The reaction of UF_6_ with gaseous ammonia should also lead to the formation of NH_4_UF_6_.^14,15^ By reacting dry nitrates with nitrosyl hexafluoridouranate­(V), NOUF_6_, hexafluoridouaranates­(V) can also be obtained; see reaction eq 2.^4,16^

$$\begin{array}{l} {\mathrm{NOUF}}_{6\left(s\right)}+{M\mathrm{NO}}_{3\left(s\right)}\underset{\Delta T}{\to }{M\mathrm{UF}}_{6\left(s\right)}+2{\mathrm{NO}}_{2\left(g\right)}\\ M=\mathrm{Li}-\mathrm{Cs} \end{array}$$ 2

We report here on the preparation of previously described alkali metal hexafluoridouranates­(V), but here for the first time in single-crystalline form, and on a comprehensive structural-chemical study of these. We shed light on the structural peculiarities of previous crystal structure reports of some of these compounds.

## Results and Discussion

### Preparation of Alkali Metal Hexafluoridouranates­(V)

The homologous series of alkali metal hexafluoridouranates­(V) can be easily prepared in solution due to the high solubility of alkali metal fluorides in aHF, which has already been reported in the literature for these salts.^3,17–19^ The formation can be described with reaction eq 3, and further details are given in the Experimental Section.

$$\begin{array}{l} {\mathrm{UF}}_{5\left(s\right)}+{MF}_{\left(\mathrm{solv}\right)}\underset{\mathrm{aHF}\left(l\right)}{\to }{M\mathrm{UF}}_{6\left(\mathrm{solv}\right)}\\ M=\mathrm{Li}-\mathrm{Cs} \end{array}$$ 3

Photographs of the compounds as well as their powder X-ray diffraction patterns are given in the Supporting Information together with a comparison with diffraction patterns from the literature and those calculated from our structure models based on single-crystal X-ray diffraction.

### Preparation of Silver­(I) Hexafluoridouranate­(V)

During attempts to synthesize a silver­(I) carbonyl compound of the putative composition [Ag­(CO)_2_]­[UF_6_] from the oxidation of Ag powder with UF_6_ in aHF under CO atmosphere,^20^ we obtained single crystals of AgUF_6_ as a side product. The reaction equation is shown in eq 4. Further details are given in the Experimental Section.

4 $$\mathrm{Ag}+{\mathrm{UF}}_{6}\underset{\mathrm{aHF}}{\to }{\mathrm{AgUF}}_{6}$$

### Preparation of Thallium­(I) Hexafluoridouranate­(V)

Thallium­(I) hexafluoridouranate­(V), TlUF_6_, was obtained in a solid-state reaction of TlF with UF_5_.^21^ Due to its good solubility in aHF, TlUF_6_ was prepared in analogy to the alkali metal hexafluoridouranates­(V);^18^ see reaction eq 3. For further details, see the Experimental Section.

### Preparation of Oxonium Hexafluoridouranate­(V)

Previous studies have described the compounds HUF_6_·1.25H_2_O and HUF_6_·2.5H_2_O, which crystallize on cooling from hydrofluoric acid solution saturated with UF_5_ (48% HF).^22^ The reaction of equimolar amounts of water and UF_5_ in hydrogen fluoride leads to the formation of oxonium hexafluoridouranate­(V), H_3_OUF_6_.^23^ It has been speculated that the former compounds may represent H_3_OUF_6_ hydrates.^4^

We therefore investigated whether the solvolysis of silicon dioxide in hydrogen fluoride in the presence of β-uranium­(V) fluoride leads to the formation of H_3_OUF_6_. The rate-determining step should be the solvolysis in which solvated oxonium fluoride and silicon tetrafluoride are formed; see reaction eq 5.

$${\mathrm{SiO}}_{2\left(s\right)}+6{\mathrm{HF}}_{\left(l\right)}\underset{{\mathrm{HF}}_{\left(l\right)}\;}{\to }2H_{3}{\mathrm{OF}}_{\left(\mathrm{solv}\right)}+{\mathrm{SiF}}_{4\left(g\right)}$$ 5

The oxonium fluoride now acts as a fluoride ion donor for the Lewis acid UF_5_ and the solvated H_3_OUF_6_ is formed, as shown in reaction eq 6.

$${\mathrm{UF}}_{5\left(s\right)}+H_{3}{\mathrm{OF}}_{\left(\mathrm{solv}\right)}\underset{{\mathrm{HF}}_{\left(l\right)}\;}{\to }H_{3}{\mathrm{OUF}}_{6\left(\mathrm{solv}\right)}$$ 6

In three reaction batches, different forms of silicon dioxide (amorphous, annealed, and silica glass wool) were reacted with β-UF_5_ in a 1:2 ratio in hydrogen fluoride in FEP Schlenk tubes at room temperature. The reaction mixture with amorphous SiO_2_ showed a slight blue coloration of the solution and an inhomogeneous sediment after 4 days at room temperature. After a reaction time of 3 weeks, annealed SiO_2_ resulted in a weak blue solution with a gray, inhomogeneous sediment. The preparation with silica glass wool showed no sediment after 3 days of reaction, and the solution was blue in color. The slow removal of the hydrogen fluoride in a vacuum led to a blue-green homogeneous reaction product, which corresponds to the description of H_3_OUF_6_ in the literature.^23^ A photograph of the product received is shown in Figure S3. Among the reactants used, silica glass wool is, therefore, best suited for the synthesis of the oxonium salt.

### Preparation of Barium Dodecafluoridodiuranate­(V)–Hydrogen Fluoride­(1/1.36)

The crystal structure of the compound Sr­[U_2_F_12_] was already determined in the past.^24^ Similar to this, we synthesized barium dodecafluoridodiuranate­(V)–hydrogen fluoride­(1/1.36), Ba­[U_2_F_12_]·1.36HF, by reacting BaF_2_ with UF_5_ in aHF according to eq 7. Further details are given in the Experimental Section.

$$2{\mathrm{UF}}_{5}+{\mathrm{BaF}}_{2}+\mathrm{HF}\underset{\mathrm{aHF}}{\to }\mathrm{Ba}[U_{2}F_{12}]\cdot \mathrm{HF}$$ 7

### The Single-Crystal Structure of Lithium Hexafluoridouranate­(V), LiUF_6_

Obtaining suitable crystals for structure determination on the single-crystal diffractometer proved to be difficult for lithium hexafluoridouranate­(V), as it has the lowest solubility reported among the alkali metal hexafluoridouranates­(V) in aHF.^17^ Attempts to recrystallize microcrystalline LiUF_6_ from aHF, acetonitrile, as well as sulfur dioxide failed. In none of the solvents could a noticeable solubility, associated with a coloration of the solution or crystal growth, be observed. The resolvation thus seems to be strongly inhibited.

Single-crystalline LiUF_6_ of light blue color was finally obtained from a synthesis with a comparatively large amount of aHF (10 mL) as the solvent, in which it was distilled very slowly over several hours in a vacuum into a separate FEP Schlenk tube. Selected crystallographic data and details of the structure determination are given in Table S2, and atomic coordinates, Wyckoff positions, site symmetries, and isotropic and anisotropic displacement parameters are reported in Tables S3 and S4 in the Supporting Information.

LiUF_6_ crystallizes in the LiSbF_6_ structure type (*hR*24). Figure 1 shows a section of the crystal structure; selected atom distances and angles are given in Table 1.

**Figure 1:**
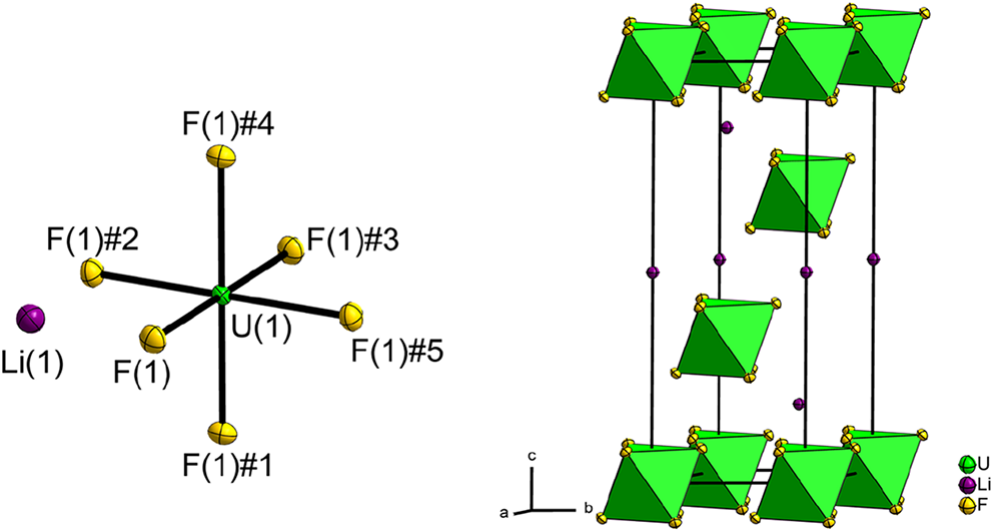
Section of the crystal structure of LiUF_6_ on the left, crystal structure of LiUF_6_ with coordination polyhedra for the UF_6_^–^ ions in green. Displacement ellipsoids at 70% probability at 100 K. Symmetry transformations for the generation of equivalent atoms: #1 1 – *y*, *x* – *y*, *z*; #2 1 – *x* + *y*, 1 – *x*, *z*; #3 4/3 −*x*, 2/3 – *y*, 2/3 – *z*; #4 1/3 + *y*, 2/3 – *x* + *y*, 2/3 – *z*; #5 1/3 + *x* – *y*, −1/3 + *x*, 2/3 – *z*.

**Table 1:** Selected Atom Distances *d* and Angles ∠_XYZ_ of LiUF_6_

atom distance *d*/Å		angle ∠_XYZ_/°	
U(1)–F(1)	2.0624(2)	F–U–F	89.774(1)–90.218(1)
Li(1)–F(1)	2.0061(2)	F–U–F	179.964(1)
U(1)–U(1)#1	5.1902(7)		

The uranium atom U(1) on Wyckoff position 3*a* (3̅.) is coordinated in its first sphere by fluorine atom F(1) and its five symmetry-equivalents F(1)#1–F(1)#5 (Figure 1). Overall, the molecular hexafluoridothiocyanate (V) ion UF_6_^–^ is formed. Figure 1 shows the formed coordination polyhedron and crystal structure. Due to the position of the uranium atom, the UF_6_^–^ ion cannot have the ideal octahedral symmetry of *O*_*h*_, but only that of the subgroup *S*_6_ (≡*C*_3*i*_).^25,26^ This corresponds to a distortion along the *C*_3_ axes of an octahedron. The deviation from the ideal values is also reflected in the angles formed between the fluorine and uranium atoms, which lie in the range of 89.774(1)–90.218(1)° and axially at 179.964(1)°. The tendency of distortion of the UF_6_^–^ ion has already been observed in single-crystal structure studies of other representatives of the compound class such as nitrosyl hexafluoridouranate­(V), NOUF_6_, and cesium hexafluoridouranate­(V), CsUF_6_.^13,27^

The atomic distance U(1)–F(1) is 2.0624(2) Å, which is equal within the tripled standard uncertainty to the values of NOUF_6_, where a U–F distance of 2.0671(9) Å (*T* = 100 K) was reported.^13^ It also agrees with CsUF_6_ with a U–F distance of 2.057(6) Å (*T* not reported) and with the average U–F distances in bis­(triphenylphosphonium)­iminium hexafluoridouranate­(V), (PPN)­UF_6_, with 2.03(2) Å (*T* not reported).^27,28^ In relation to binary uranium fluorides with U atoms in coordination number 6, α–UF_5_, _∞_^1^[UF_4/1_F_2/2_], with a U–F distance of 2.020(5) and a μ-F–U distance of 2.236(1) Å (*T* not reported), and in UF_6_ with 2.023(6) Å (*T* = 77 K), the distances in the UF_6_^–^ salts are slightly increased due to the negative charge of the anion.^29,30^

Around the lithium atom Li(1) (3*b*, 3̅.), also an octahedron-like coordination polyhedron with the fluorine atoms is formed.^31^ The atomic distance Li(1)–F(1) of 2.0061(2) Å is shorter than in LiF with 2.013 Å (*T* = 298 K).^32^

The coordination polyhedra of lithium and uranium atoms are oriented differently. One coordination polyhedron is surrounded by six polyhedra of the other cation type. The crystal structure can also be described as a sequence of alternating coordination polyhedron layers. When looking at such UF_6_^–^ layers, a stacking sequence of |:ABC:| is observed, corresponding to Jagodzinski symbol *c*.^25^ The stacking is therefore related to the cubic close packing.

### The Single-Crystal Structure of Sodium Hexafluoridouranate­(V), NaUF_6_—Rhombohedral Modification

The sodium salt of alkali metal hexafluoridouranate­(V), NaUF_6_, is the only one in the series to have been described as polymorphic to date.^5,17^ Two modifications, one cubic and one rhombohedral, were reported. In the preparation of NaF and UF_5_ in hydrogen fluoride, the distillation rate of the solvent has an influence on the modification of the phase obtained. A high rate favors the formation of the higher-symmetry modification, while a low rate favors the formation of the lower-symmetry modification.^17^ The temperature also has an influence on the modification formed. Ostwald’s step rule,^25,33,34^ the phenomenon that a metastable modification of a compound crystallizes first, which then transforms into a thermodynamically more favorable modification, seems to be also fulfilled here.

Blueish crystals of NaUF_6_ were obtained by using a low removal rate of aHF at room temperature from the reaction mixture. NaUF_6_ crystallizes, like LiUF_6_, in the LiSbF_6_ structure type, and no detailed structural description will be given. Selected crystallographic data and details of the structure determination are given in Tables S5–S7. Selected atom distances and angles are given in Table 2.

**Table 2:** Selected Atom Distances *d* and Angles ∠_XYZ_ of NaUF_6_ (Rhombohedral Polymorph)

atom distance *d*/Å		angle ∠_XYZ_/°	
U(1)–F(1)	2.0661(7)	F–U–F	89.563(2)–90.437(2)
Na(1)–F(1)	2.2768(7)	F–U–F	179.989(2); 179.985; 180.00
U(1)–U(1)	6.1078(9)		

The U–F distance is 2.0661(7) Å and is slightly larger than that in LiUF_6_. The formed octahedron-like coordination polyhedron, with angles in the range 89.563(2)–90.437(2)° and three different axial angles, shows a larger distortion than the UF_6_^–^ ion in LiUF_6_.

The sodium atoms are coordinated by six fluorine atoms at a distance of 2.2768(7) Å in the shape of an octahedron. The Na–F distance is comparable to that in NaF, which is 2.315 Å (*T* = 273 K).^35^ Compared to the lithium salt, the coordination polyhedra have a different orientation, which can be explained by the stronger distortion of the anions. This is probably due to the larger effective ionic radius of Na^+^ with 102 pm for the coordination number six in relation to Li^+^ with 76 pm.^36^

### The Single-Crystal Structure of Potassium Hexafluoridouranate­(V), KUF_6_

Putative space groups and lattice parameters have already been reported for potassium hexafluoridouranate­(V), KUF_6_, but single-crystal structure studies have not yet been carried out. A first study assigned the orthorhombic crystal system with lattice parameters *a* = 5.73, *b* = 5.6, and *c* = 3.98 Å, *V* = 127.7 Å^3^. *Pccm* (No. 49) was named as a possible space group.^37^ Another publication also proposed the orthorhombic crystal system with lattice parameters *a* = 5.61, *b* = 11.46, and *c* = 7.96 Å, *V* = 511.8 Å^3^, a quadrupled volume compared to the previous one, with the possible space groups *Cmme* (No. 67) or *Aem*2 (No. 39).^38^ In the course of a single-crystal structure investigation of rubidium hexafluoridoprotactinate­(V), RbPaF_6_, space group *Cmme*, KUF_6_ was described as isotypic due to its similar lattice parameters.^38–40^

Blueish crystals of KUF_6_ were obtained at room temperature and subjected to single-crystal X-ray analysis. Initially, the structure solutions and refinements were attempted in the orthorhombic crystal system with the lattice parameters *a* = 11.442(2), *b* = 8.0345(1), and *c* = 5.5655(1) Å, *V* = 519.87 Å^3^ in space groups *Cmme* and *Aem2*. However, the refinements did not lead to any satisfactory structural model, as the displacement parameters showed unusual values. The successive descent into corresponding nonisomorphic orthorhombic subgroups and the application of twin laws showed no improvement of the structural models.^31,41^ Finally, a proper structural model in the monoclinic subgroup *C*2/*m* (No. 12, *mS*32) of space group *Cmme* was obtained with the lattice parameters *a* = 11.442(2), *b* = 8.0345(1), *c* = 5.5655(1) Å, β = 90.138(9)°, *V* = 511.62(4) Å^3^, *Z* = 4 at *T* = 100 K.^31^ Selected crystallographic data and details of the structure determination are given in Tables S8–S10 in the Supporting Information. A section of the crystal structure of KUF_6_ is shown in Figure 2. Selected atom distances are listed in Table 3.

**Table 3:** Selected Atom Distances *d* of KUF_6_

Atom distances *d*/Å			
U(1)–F(1)	2.0633(3)	K(1)–F(1)	2.6995(3)
U(1)–F(2)	2.0635(3)	K(1)–F(1)	3.0950(3)
U(1)–F(3)	2.3400(2)	K(1)–F(2)	2.6897(3)
U(1)–F(4)	2.3403(2)	K(1)–F(2)	3.0902(3)
U(1)–U(1)#1	4.0102(5)	K(1)–F(3)	2.6762(4)
U(1)–U(1)#2	4.0243(6)	K(1)–F(4)	2.6886(4)

**Figure 2:**
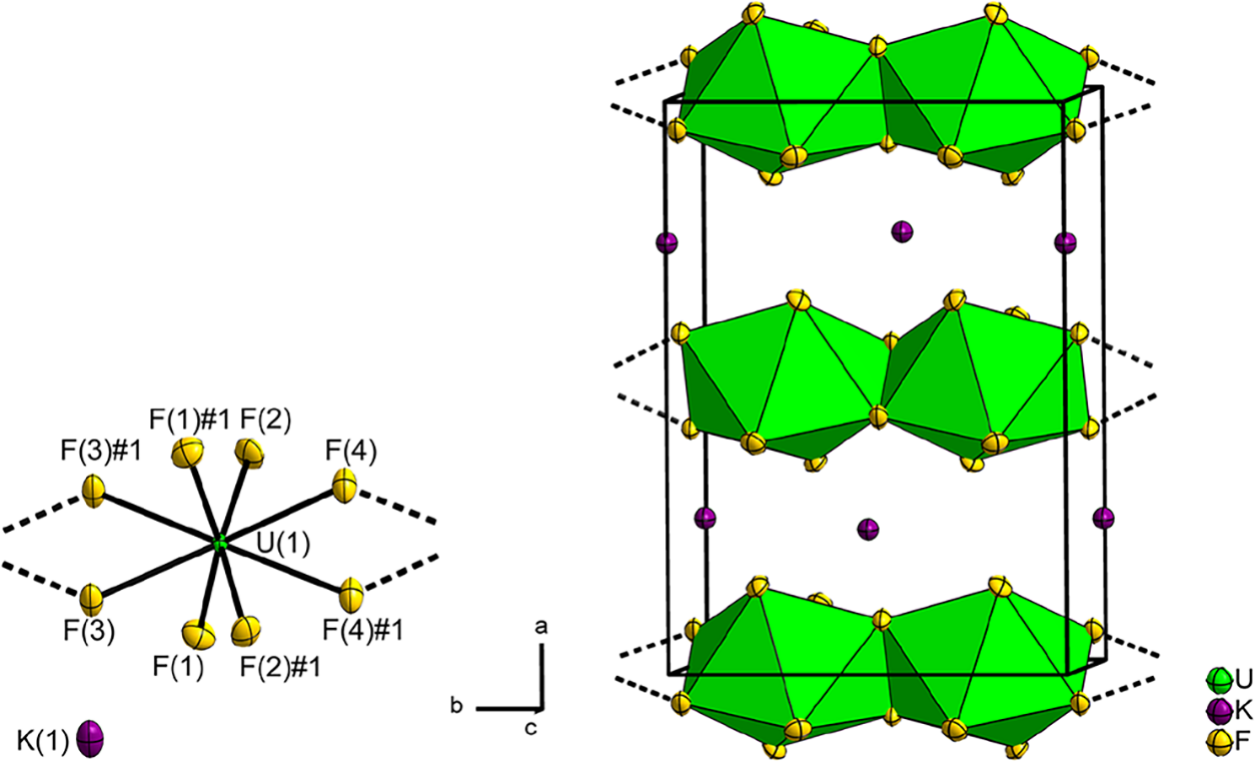
Section of the crystal structure of KUF_6_ on the left; on the right, the crystal structure of KUF_6_, displacement ellipsoids at 70% probability at 100 K. The first coordination spheres of the U atoms are shown as green coordination polyhedra. Displacement ellipsoids at 70% probability at 100 K. Symmetry transformations for the generation of equivalent atoms: F(1)#1 1 – *y*, *x* – *y*, *z*; F(2)#1 1 – *y*, *x* – *y*, *z*; F(3)#1 1 – *y*, *x* – *y*, *z*; F(4)#1 1 – *y*, *x* – *y*, *z*.

The asymmetric unit contains a uranium atom U(1), which is located at the Wyckoff position 4*h* with site symmetry 2.^26,31^ In the first sphere, the U atom is coordinated by eight fluorine atoms. Of these, the fluorine atoms F(1) and F(2) occupy the Wyckoff position 8*j* and the fluorine atoms F(3) and F(4) the position 4*i*. The isopointal fluorine atoms each lie on different crystallographic orbits. The equivalent fluorine atoms F(1)#1–F(4)#1 are generated by symmetry transformations; for these, see the caption of Figure 2. The uranium atom is thus coordinated by the fluorine atoms in the form of a distorted trigonal dodecahedron. The ideal polyhedron is also known as the Johnson solid J_84_ and has the symmetry *D*_2*d*_.^42^ The edges formed by the atoms F(3) and F(3)#1 and by F(4) and F(4)#1 are joined by further coordination polyhedra, so that a one-dimensional infinite chain is formed, which can be described using the Niggli notation _∞_^1^{[UF_4/1_F_4/2_]^−^}.^43^ The chain is aligned parallel to the crystallographic *b* axis, see Figure 2. Such a structural motif of the anions can also be observed in other fluorides. For example, in RbPaF_6_, previously assumed to be isotypic, in tetramethylammonium hexafluoridoprotactinate­(V), (N­(CH_3_)_4_)­PaF_6_, and in potassium hexafluoridozirconate­(IV) and -hafnate­(IV), K_2_ZrF_6_ and K_2_HfF_6_.^44,45^

Looking at the coordination sphere around the uranium atom at a distance of 4.10 to 4.25 Å, the fluorine atoms form a distorted gyrobifastigium with corner and edge linkages. The ideal polyhedron is also known as a twisted double wedge, has the symmetry *D*_2*d*_ and also belongs to the Johnson solids with the number J_26_.^42^ Gyrobifastigium is the only Johnson solid that can completely fill the three-dimensional (3D) space.^46^

In KUF_6_, two different distances between the uranium atom and the fluorine atoms can be observed: a short distance with 2.0633(3) Å for the terminally bound fluorine atoms and a larger one with 2.3400(2) Å for the bridging fluorine atoms. The former is comparable to those in simple UF_6_^–^ salts with molecular anions. The distances for the bridging fluorine atoms in the _∞_^1^{[UF_4/1_F_4/2_]^−^} anion are slightly larger, as expected, compared to those in the electrically neutral α-UF_5_ at 2.236(1) Å (*T* not reported), β-UF_5_ (_∞_^3^[UF_2/1_F_6/2_]) at 2.27(2) Å (*T* = r.t.), and the hydrogen cyanide adduct _∞_^1^[UF_4/1_F_2/2_(HCN)_2/1_] with 2.227(6)–2.330(5) Å (*T* = 100 K).^29,47,48^

The distances of the uranium atoms within the chains of 4.0102(5) and 4.0243(6) Å are quite small compared to those of hexafluoridouranates­(V) investigated so far. In NOUF_6_, U–U distances of 5.1732(2) Å (*T* = 100 K) are observed, in CsUF_6_ of 5.417(3) Å (*T* not reported).^13,27^ Magnetochemical investigations were carried out on the latter two, but no coupling of the magnetic centers was observed there.^13,49^ However, an interaction could occur in KUF_6_ due to the smaller distance.

The potassium atom K(1) is located at Wyckoff position 4*i* with site symmetry *m*.^31^ It is coordinated by six fluorine atoms with a smaller distance of approximately 2.68 Å and four with a larger distance of ca. 3.09 Å. Of these, two are terminally bound with distances of 2.6762(4) Å for F(3) and 2.6886(4) Å for F(4), which are comparable to those in KF with 2.664 Å (*T* not reported).^50^ The remaining fluorine atoms are bridging, so that one-dimensional infinite chains are obtained in which two opposite quadrilateral faces are linked. A distorted pentagonal prism is thus obtained as a coordination polyhedron in which, compared with the ideal body, one fluorine atom is displaced out of the plane on each of the two pentagonal faces.

### The Single-Crystal Structure of Rubidium Hexafluoridouranate­(V), RbUF_6_

For rubidium hexafluoridouranate­(V), RbUF_6_, similar speculations were made for the possible space group as for KUF_6_, see above.^17,37,40^ Blueish-green crystals were obtained and analyzed by X-ray diffraction. Due to the similar lattice parameters and comparable effective ionic radii of the Rb^+^ and K^+^ ions, the compound crystallizes in the monoclinic crystal system with *Z* = 4 in space group *C*2/*m* (No. 12).^36^ Selected crystallographic data and details of the structure determination are given in Tables S11–S13 in the Supporting Information.

RbUF_6_ is isotypic to KUF_6_ so that no detailed structural discussion will be made. Selected atom distances are listed in Table 4.

**Table 4:** Selected Atom Distances *d* of RbUF_6_

atom distances *d*/Å			
U(1)–F(1)	2.0600(4)	Rb(1)–F(1)	2.833(4)
U(1)–F(2)	2.0573(4)	Rb(1)–F(1)	3.1123(4)
U(1)–F(3)	2.3286(2)	Rb(1)–F(2)	2.8308(4)
U(1)–F(4)	2.3286(2)	Rb(1)–F(2)	3.1168(4)
U(1)–U(1)#1	4.0019(9)	Rb(1)–F(3)	2.7691(5)
U(1)–U(1)#2	4.0148(9)	Rb(1)–F(4)	2.7694(5)

The uranium atom U(1) is coordinated by fluorine atoms at a distance of approximately 2.057(4) and 2.3286(2) Å, forming a distorted trigonal dodecahedron as a coordination polyhedron. The measured distances show a slight shortening compared to KUF_6_, which is probably due to the larger Rb^+^ ion and a shortening of the U–F bond.^36^ The U–U distances within the chains are also slightly smaller at 4.0019(9) and 4.0148(9) Å.

The rubidium atoms are coordinated by 10 fluorine atoms in the first coordination sphere, resulting in a distorted pentagonal prism as the coordination polyhedron. Smaller Rb–F distances of about 2.8 Å and larger distances of about 3.1 Å are observed. The two terminally bound fluorine atoms, F(3) with 2.7691(5) Å and F(4) with 2.7694(5) Å, have a comparable Rb–F distance as in RbF, 2.827 Å (*T* not reported).^51^

### The Single-Crystal Structure of Silver Hexafluoridouranate­(V), AgUF_6_

During studies of the formation of silver carbonyl compounds, we obtained yellowish needle-shaped crystals of AgUF_6_. Silver hexafluoridouranate­(V) was previously reported to crystallize in space group *P*4_2_/*mcm*, which was established based on powder X-ray diffraction patterns.^37,52^ However, our single-crystal X-ray diffraction study showed that AgUF_6_ crystallizes in the CaTbF_6_ structure type in the tetragonal space group *P*4_2_/*m* (No. 84, *tP*16) with the lattice parameters *a* = 5.4188(2), *c* = 7.9458(4) Å, *V* = 233.32(2) Å^3^, *Z* = 2 at *T* = 100 K.^31^ Selected crystallographic data and details of the structure determination are given in Tables S14–S16 in the Supporting Information. A section of the crystal structure of AgUF_6_ is shown in Figure 3. Selected atom distances are given in Table 5.

**Table 5:** Selected Atom Distances *d* of AgUF_6_

atom distances *d*/Å			
U(1)–F(1)	2.3292(5)	Ag(1)–F(1)	2.6499(5)
U(1)–F(2)	2.0641(7)	Ag(1)–F(2)	2.4345(8)
U(1)–U(1)	3.9729(2)		

**Figure 3:**
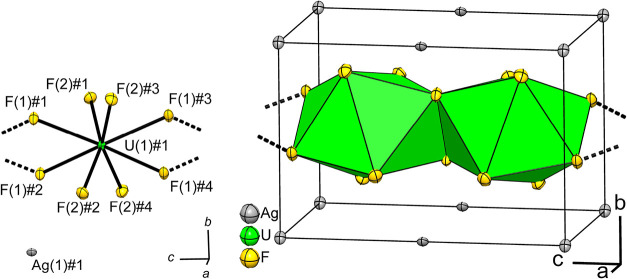
Section of the crystal structure of AgUF_6_ on the left; on the right, the crystal structure of AgUF_6_. The first coordination spheres of the U atoms are shown as green coordination polyhedra. Displacement ellipsoids at 70% probability at 100 K. Symmetry transformations for the generation of equivalent atoms: Ag(1)#1 1 + *x*, *y*, *z*; U(1)#1 1 – *y*, *x*, −1/2 + *z*; F(1)#1 *y*, 1 – *x*, −1/2 + *z*; F(1)#2 1 – *y*, *x*, −1/2 + *z*; F(1)#3 x, *y*, −1 + *z*; F(1)#4 1 – *x*, 1 – *y*, −1 + *z*; F(2)#1 *–* *x*, 1 – *y*, *z*; F(2)#2 1 + *x*, *y*, *z*; F(2)#3 1 – *y*, 1 + *x*, 1/2 – *z*; F(2)#4 *y*, −*x*, 1/2 – *z*.

The asymmetric unit contains a uranium atom U(1), which is located at the Wyckoff position 2*f* (4̅.).^26,31^ In the first sphere, the U atom is coordinated by eight fluorine atoms. Of these, the fluorine atom F(1) occupies Wyckoff position 4*j* (*m*..) and fluorine atom F(2) the position 8*k* (1). The equivalent fluorine atoms F(1)#1 to F(2)#4 are generated by symmetry transformations; for these, see the caption of Figure 3. Since the coordination sphere of the uranium atom is similar to those in KUF_6_ and RbUF_6_, we refrain from a detailed discussion here.

The silver atom Ag(1) is located at Wyckoff position 2*a* with site symmetry 2/*m*...^31^ It is coordinated by four fluorine atoms with a smaller distance of 2.4345(8) Å and two with a larger distance of 2.6499(5) Å in the shape of a distorted octahedron. The coordination polyhedra of the silver atoms are connected via shared corners to the coordination polyhedra of the uranium atoms. The packing of the octahedra along the crystallographic *a* and *c* axes is shown in Figure 4. If Ag–F distances of up to 3.1 Å are considered, after which a larger gap comes, then a distorted pentagonal antiprism results.

**Figure 4:**
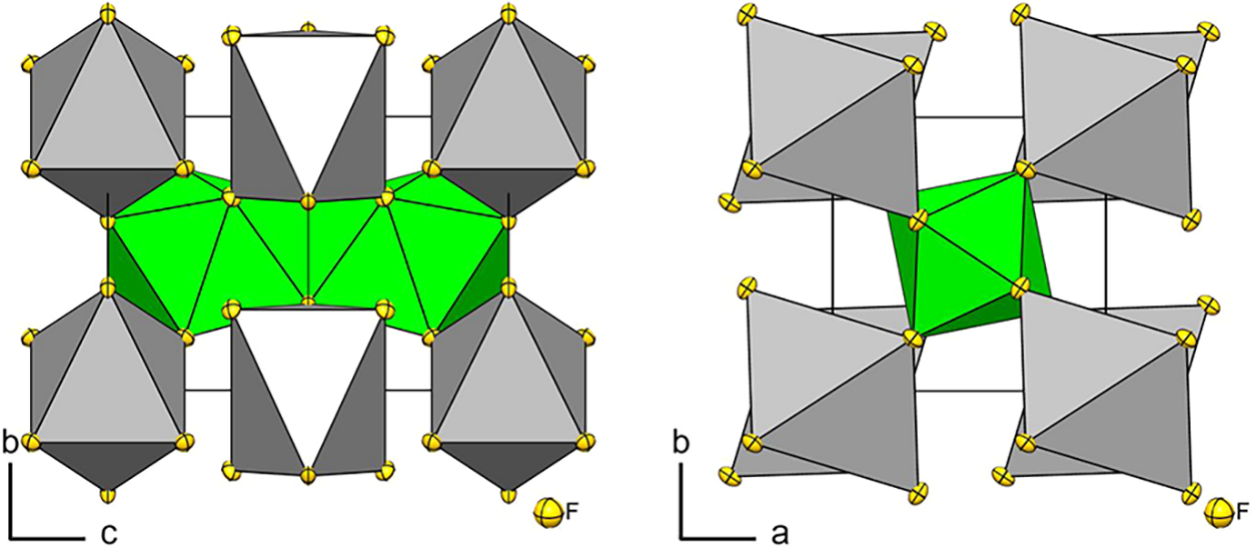
Sections of the crystal structure of AgUF_6_. The packing of the coordination polyhedra of Ag is shown in gray, and those of U in green along the crystallographic *a* and *c* axes. Displacement ellipsoids at 70% probability at 100 K.

In the previously proposed structure model in space group *P*4_2_/*mcm*, the coordination numbers of the U atoms were six, and those of the Ag atoms were eight with octahedral and square-antiprismatic coordination spheres. As said above, our structure model shows a coordination number of eight for the U atoms in the shape of a distorted trigonal dodecahedron as in the respective K and Rb compounds. The coordination number of the Ag^+^ cation is six in the shape of a distorted octahedron or ten in the shape of a distorted pentagonal antiprism, considering Ag^–^ distances up to 3.1 Å. The coordination numbers of the K^+^ and the Rb^+^ cations of the UF_6_^–^ salts are also 10; however, a distorted pentagonal prism is observed. So, the difference in coordination spheres is where the different crystal structures of AgUF_6_ compared to KUF_6_ and RbUF_6_ come from.

### The Single-Crystal Structure of Thallium­(I) Hexafluoridouranate­(V), TlUF_6_

In the literature, thallium hexafluoridouranate­(V), TlUF_6_, has been assigned several possible space groups, similar to the cases of KUF_6_ and RbUF_6_ described above. However, a single-crystal structure investigation has not yet been reported.^38–40^ Blue-green crystals were obtained and investigated by X-ray diffraction. Due to the cell parameters being similar to KUF_6_ and RbUF_6_, the structure model was refined in space group *C*2/*m* (No. 12). Selected crystallographic data and details of the structure determination are given in Tables S14–S16 in the Supporting Information.

TlUF_6_ is isotypic to KUF_6_ so that no detailed structural discussion will be made. Selected atom distances are given in Table 6. The uranium atom U(1) is coordinated at distances of 2.05 to 2.32 Å by a total of eight fluorine atoms in the shape of a distorted trigonal dodecahedron. In relation to KUF_6_, the terminal and bridging fluorine atoms, like in RbUF_6_, have smaller distances to the uranium atom, which can be explained by the larger effective ionic radius of the Tl^+^ ion and the resulting shortening of the U–F bond.^36^ This influence can also be observed in the U–U distances within the chain, which are the smallest in the series at 3.9937(9) and 4.0046(9) Å.

**Table 6:** Selected Atom Distances *d* of TlUF_6_

atom distances *d*/Å			
U(1)–F(1)	2.0538(4)	Tl(1)–F(1)	2.8394(4)
U(1)–F(2)	2.0611(4)	Tl(1)–F(1)	3.1510(3)
U(1)–F(3)	2.3241(3)	Tl(1)–F(2)	2.8311(4)
U(1)–F(4)	2.3264(3)	Tl(1)–F(2)	3.1485(4)
U(1)–U(1)#1	3.9937(9)	Tl(1)–F(3)	2.7962(5)
U(1)–U(1)#2	4.0046(9)	Tl(1)–F(4)	2.8052(6)

As in KUF_6_ and RbUF_6_, the Tl atoms are coordinated by ten F atoms in the first coordination sphere, resulting in a distorted pentagonal prism as a polyhedron. Smaller Tl–F distances of approximately 2.82 Å and larger ones of ca. 3.15 Å are observed. The two terminally bound fluorine atoms, F(3) with 2.7962(5) Å and F(4) with 2.8052(6) Å, have a comparable Tl–F distance as in TlF, 2.590–3.040 Å (*T* not reported).^53^

### The Single-Crystal Structure of Oxonium Hexafluoridouranate­(V), H_3_OUF_6_

So far, only the crystal system of the oxonium salt H_3_OUF_6_ had been determined.^23^ It was described as cubic with the lattice parameter *a* = 5.2229(5) Å with the volume *V* = 142.47 Å^3^ (*T* = r.t.). On the basis of the powder X-ray diffractograms, however, it was not possible to distinguish whether the compound crystallizes cubic primitive, such as NOUF_7_, or body-centered cubic, such as NOUF_6_.^23,54^

Blueish crystals of H_3_OUF_6_ were obtained and investigated by X-ray diffraction, indicating the space group *Ia*3̅ (No. 206). Selected crystallographic data and details of the structure determination are given in Tables S17–S19 in the Supporting Information. The hydrogen atoms could not be located due to the site symmetry. A section of the crystal structure is shown in Figure 5, and selected atom distances and angles are given in Table 7.

**Figure 5:**
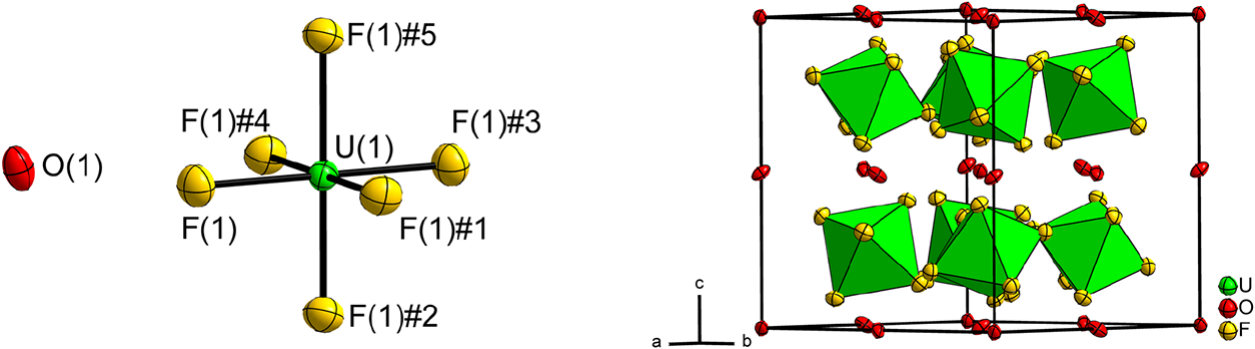
Section of the crystal structure of H_3_OUF_6_ on the left; crystal structure of H_3_OUF_6_ with green coordination polyhedra for the UF_6_^–^ ions on the right. Displacement ellipsoids with 70% probability at 100 K. Symmetry transformations for the generation of equivalent atoms: #1 1 – *y*, −1/2 + *z*, 3/2 – *x*; #2 3/2 – *z*, 1 – *x*, 1/2 + *y*; #3 3/2 – *x*, 1/2 – *y*, 3/2 – *z*; #4 1/2 + *y*, 1 – *z*, *x*; #5 *z*, −1/2 + *x*, 1 – *y*.

**Table 7:** Selected Atom Distances *d* and Angles ∠_XYZ_ of H_3_OUF_6_

atom distances *d*/Å		angles ∠_XYZ_/°	
U(1)–F(1)	2.0545(4)	F–U–F	89.183(2); 90.817(2)
O(1)–F(1)	2.6378(4)	F–U–F	179.982(2); 180.00; 180.00
U(1)–U(1)	5.1752(6)		

The uranium atom U(1) resides on Wyckoff position 8*b* (.3̅.).^31^ At a distance of 2.0545(4) Å from this, the fluorine atom F(1) is located at general position 48*e*. Symmetry transformations generate the equivalent fluorine atoms F(1)#1 to F(1)#5, whereby the uranium atom is coordinated by six fluorine atoms in an octahedron-like manner; see Figure 5. Consistent with the site symmetry of the U(1) atom, the coordination polyhedron exhibits distortions compared to the ideal octahedron, as indicated by the F–U–F angles of 89.183(2) and 90.817(2)° and the axial angle of 179.982(2)°, in addition to the 180° angles. The distances and angles in the UF_6_^–^ ion are comparable to those in LiUF_6_ and NaUF_6_, see above.

The oxygen atom O(1) resides on Wyckoff position 8*a* (.3̅.).^31^ The hydrogen atom positions could not be determined due to the special position of the oxygen atom. However, the presence of the oxonium ion could be confirmed by IR spectroscopy; see Figure 6, where the O–H band is broadened and shifted due to hydrogen bonding (3268, 2808 cm^–1^). The bands at 1617 and 957 cm^–1^ can also be assigned to the H_3_O^+^ cation.^23,55,56^ The band at 485 cm^–1^ is due to the UF_6_^–^ anion.^23^ From the crystal structure, no precise statements can be made about hydrogen bonds of the type O–H···F. Comparing the donor···acceptor distances, O···F, of 2.6378(4) Å with those of other oxonium salts in which hydrogen bonding has been observed by X-ray diffraction, such as H_3_OAsF_6_, 2.6691(3) Å (*T* = 238 K), H_3_OSb_2_F_11_, 2.553(8) to 2.903(9) Å (*T* = 294 K), or H_3_OBF_4_, 2.5771(4) to 2.6090(4) Å (*T* = 247 K), the presence of these in H_3_OUF_6_ is nevertheless quite probable and proven by the IR spectrum.^57–59^

**Figure 6:**
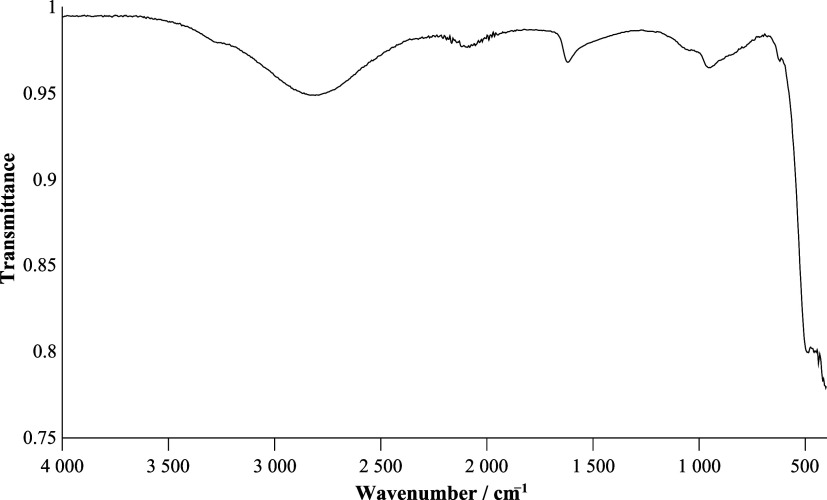
ATR-IR spectrum of H_3_OUF_6_ recorded at room temperature with 32 scans and a resolution of 4 cm^–1^.

The lattice parameters indicate a structural relationship to O_2_PtF_6_, or NOUF_6_, in which, however, the 8*a* position is occupied by uranium atoms and the 8*b* position by the center of gravity of the cations.^13,60^ Both crystal structures are superstructures of the CsCl structure type.^13^ H_3_OUF_6_ can therefore be considered more as an anti-O_2_PtF_6_ type due to the positional parameters, related to the example of the fluorite and “antifluorite” structure type.^25^ In comparison, H_3_OSbF_6_ and potassium hexafluoridoantimonate­(V), KSbF_6_, crystallize in the same space group, similar lattice parameters and with the same occupation of the Wyckoff positions, making H_3_OUF_6_ isotypic to KSbF_6_.^55,61,62^ In the following, the structural-chemical relationship between the CsCl type and the KSbF_6_ type will be analyzed by a group–subgroup relationship with the help of a Bärnighausen tree, see Figure 7.^41,63^

**Figure 7:**
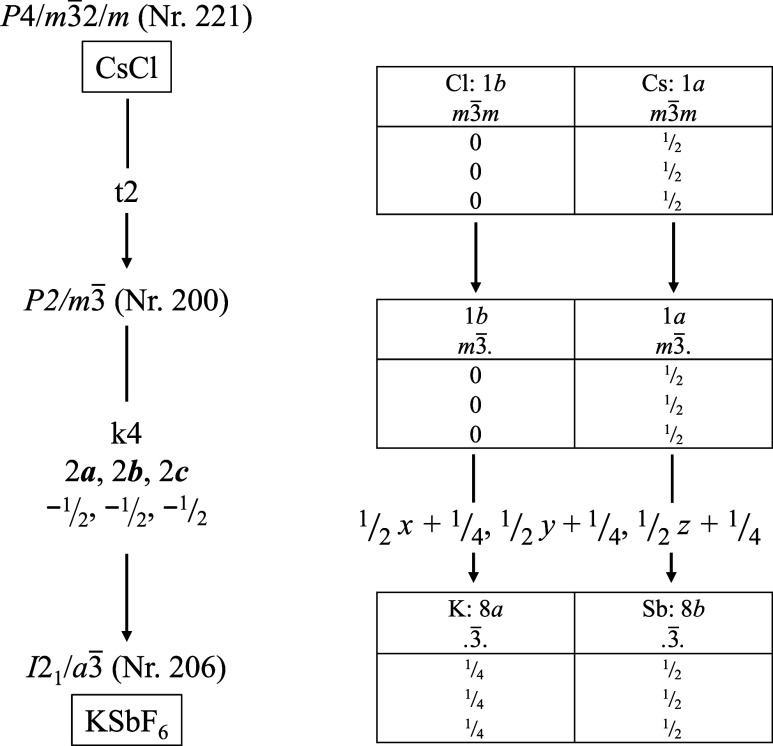
Bärnighausen tree with a group–subgroup relationship of CsCl to KSbF_6_, to which H_3_OUF_6_ is isotypic.

Starting from the aristotype CsCl, space group *P*4/*m*3̅2/*m* (No. 221), a *translationengleiche* transition of index 2 leads to subgroup *P*2/*m*3̅ (No. 200).^64,65^ This lowers the site symmetry of the 1*a* and 1*b* position from *m*3̅*m* (*O_h_* symmetry) to *m*3̅. (*T_h_* symmetry).^26,66^ Based on this subgroup, a *klassengleiche* transition of index 4 leads to space group *I*2_1_/*a*3̅ (No. 206) of hettotype KSbF_6_. In this case, the unit cell must be multiplied by a factor of 8; the Wyckoff positions develop as follows due to the origin shift of −1/2, −1/2, −1/2: 1*a* → 8*b*, 1*b* → 8*a*, which corresponds to swapping the centers of gravity of cation and anion sites. This transition reduces the site symmetries according to 3̅. (*S*_6_ symmetry).

### Powder X-ray Diffraction on Powders of the Above Compounds

The powder X-ray diffractogram of LiUF_6_ recorded at room temperature agrees well with the calculated diffractogram from the single-crystal structure analysis (*T* = 100 K), see Figure S4. A shift in the reflex positions can be attributed to the different measurement temperatures. The reflections could be indexed in the trigonal crystal system with the lattice parameters *a* = 5.27726(2) Å and *c* = 14.3676(4) Å, *V* = 346.52(2) Å^3^. The parameters are comparable to those previously reported for LiUF_6_ (ICDD entry [22–0411]) of *a* = 5.262(4), *b* = 14.295(5) Å and *V* = 342.78 Å^3^ (*T* not reported).^5^

The NaUF_6_ obtained from hydrogen fluoride solution shows reflections in the powder X-ray diffractogram recorded at room temperature that can be assigned both to the cubic modification, ICDD entry [16–0720] (*T* not reported), and to the rhombohedral modification, ICDD entry [18 1220] (*T* not reported), see Figure S5.^5,17^ A phase-pure synthesis from HF is therefore difficult, as the concentration, temperature, and distillation rate of the hydrogen fluoride have an influence on the supersaturation and polymorph formation.

KUF_6_ was previously described in the literature as orthorhombic.^38^ In the above single-crystal structure analysis, however, the monoclinic crystal system proved to be superior for a proper structure model; see above. The powder X-ray diffractogram (Figure S6), which was recorded at room temperature, is underlaid with the data of the ICDD entry [14–0661] for KUF_6_ (previous single-crystal structure) in addition to the calculated reflex positions from our single-crystal data (*T* = 100 K).^38^ The reflections could be indexed with both an orthorhombic and monoclinic cell with comparable quality factors. The orthorhombic cell has the lattice parameters *a* = 11.4785(2), *b* = 8.0181(1), and *c* = 5.6082(1) Å and *V* = 516.16(1) Å^3^, those for the monoclinic cell are *a* = 11.4856(2), *b* = 8.0187(1), *c* = 5.6118(9) Å, β = 90.08(2)°, and *V* = 516.84(2) Å^3^. Based on the single-crystal and powder X-ray data, the crystal structure of KUF_6_ can also be described as pseudo-orthorhombic. This pseudosymmetry is also evident in the diffractogram compared to the literature data set.^67^ The monoclinic unit cell differs only minimally from the orthorhombic unit cell and leads to a splitting of some reflections of the orthorhombic crystal system.

Similar to KUF_6_, an orthorhombic unit cell has been described in the literature for RbUF_6_.^5,38^ The lattice parameters were determined to be *a* = 5.82 Å, *b* = 11.89 Å, *c* = 8.03 Å, and *V* = 555.7 Å^3^ (*T* not reported).^38^ The powder X-ray diffractogram recorded at room temperature with underlaid reflex positions and intensities calculated from our single-crystal structure analysis (*T* = 100 K) and the ICDD entry [16–0748] for RbUF_6_ are shown in Figure S7.^5^ Indexing of the reflections was possible with comparable quality factors using orthorhombic and monoclinic cells, as stated above. The lattice parameters of the monoclinic cell are *a* = 11.859(3), *b* = 8.0561(1), *c* = 5.7945(3) Å, β = 90.09(3)°, and *V* = 553.6(3) Å^3^ (*T* = 298 K).

The powder X-ray diffractogram of CsUF_6_ is shown in Figure S8. The calculated reflection intensities from the crystal structure of CsUF_6_ are underlaid on the diffractogram.^27^ The lattice parameters for the single crystal were determined to be *a* = 8.0189, *c* = 8.4373 Å, and *V* = 469.85 Å^3^ (*T* not reported).^27^ An indexing of the reflections of the powder pattern also revealed a trigonal crystal system with the lattice parameters *a* = 8.0284(9), *c* = 8.4388(1) Å, and *V* = 471.05(1) Å^3^.

For thallium­(I) hexafluoridouranate­(V), similar to KUF_6_ and RbUF_6_, an orthorhombic cell has been described in the literature so far.^38–40^ The powder X-ray diffractogram recorded at room temperature is shown in Figure S9 and underlaid with the calculated reflection positions from our single-crystal structure analysis (*T* = 100 K) and the ICDD entry [16–0748] of the isotypic RbUF_6_, see above.^5^ The lattice parameters were determined as *a* = 5.956, *b* = 5.774, *c* = 4.001 Å, and *V* = 137.59 Å^3^ (*T* not reported).^68^ However, an indexing of the reflections of our powder pattern indicates a quadrupling of the unit cell, in agreement with the results of the single-crystal data.^40^ The description with orthorhombic and monoclinic cells provides comparable quality factors. The lattice parameters of the monoclinic unit cell are *a* = 11.951(3), *b* = 8.0435(2), *c* = 5.7818(2) Å, β = 89.99(3)°, and *V* = 556.0(3) Å^3^.

The powder X-ray diffractogram of H_3_OUF_6_ measured at room temperature, which is shown in Figure S10, was underlaid with the calculated reflection layers from our single-crystal structure analysis and for comparison with the ICDD entry [11–0246] of NOUF_6_ (*T* not reported).^69^ Certain similarities with H_3_OUF_6_ were attributed to the latter, but the single-crystal structure analysis has shown that the oxonium salt also crystallizes in space group *Ia*3̅ (No. 206), but NOUF_6_ is isotypic to O_2_PtF_6_ and H_3_OUF_6_ is isotypic to H_3_OSbF_6_, which corresponds to a swapping of the cation and anion sites.^13,23,61^ The extraneous reflection at approximately 28° 2θ is probably due to the reaction of H_3_OUF_6_ with the borosilicate glass. The capillary was dissolved by the oxonium salt approximately 2 days after the measurement. Ignoring the reflection, the others can be indexed in the cubic crystal system with the lattice parameters *a* = 5.2256(6) Å and *V* = 142.69(3) Å^3^. However, the single-crystal structure analysis shows an 8-fold increase in the primitive unit cell to *a* = 10.4496(1) Å and *V* = 1141.04(2) Å^3^, as was also shown for NOUF_6_.^13^

### The Single-Crystal Structure of Barium Dodecafluoridodiuranate­(V)–Hydrogen Fluoride­(1/1.36), Ba­[U_2_F_12_]·1.36HF

Greenish plate-like crystals of Ba­[U_2_F_12_]·1.36HF were obtained at −40 °C, isolated at room temperature, and subjected to single-crystal X-ray analysis. Ba­[U_2_F_12_]·1.36HF crystallizes in the orthorhombic space group *Pnma* (No. 62, *oP*68) with the lattice parameters *a* = 8.9989(8), *b* = 12.6822(9), *c* = 9.3514(6) Å, *V* = 1067.24(14) Å^3^, and *Z* = 4 at *T* = 100 K. The structure solution was also carried out in space group *Pna*2_1_, but this structure model resulted in negative displacement parameters of the fluorine atoms. Selected crystallographic data and details of the structure determination are given in Tables S20–S22 in the Supporting Information. A section of the crystal structure of Ba­[U_2_F_12_]·1.36HF is shown in Figure 8. Selected atom distances are given in Table 8.

**Table 8:** Selected Atom Distances *d* of Ba­[U_2_F_12_]·1.36HF

atom distances *d*/Å			
Ba(1)–F(2)	2.676(7)	U(1)–F(1)	2.060(7)
Ba(1)–F(3)	2.748(7)	U(1)–F(2)	2.068(7)
Ba(1)–F(4)	2.695(7)	U(1)–F(3)	2.062(7)
Ba(1)–F(5)	2.686(8)	U(1)–F(4)	2.068(7)
Ba(1)–F(7)	2.924(11)	U(1)–F(5)	2.061(7)
Ba(1)–F(8)	2.90(3)	U(1)–F(6)	2.260(7)
H(1)–F(7)	1.3(2)		

**Figure 8:**
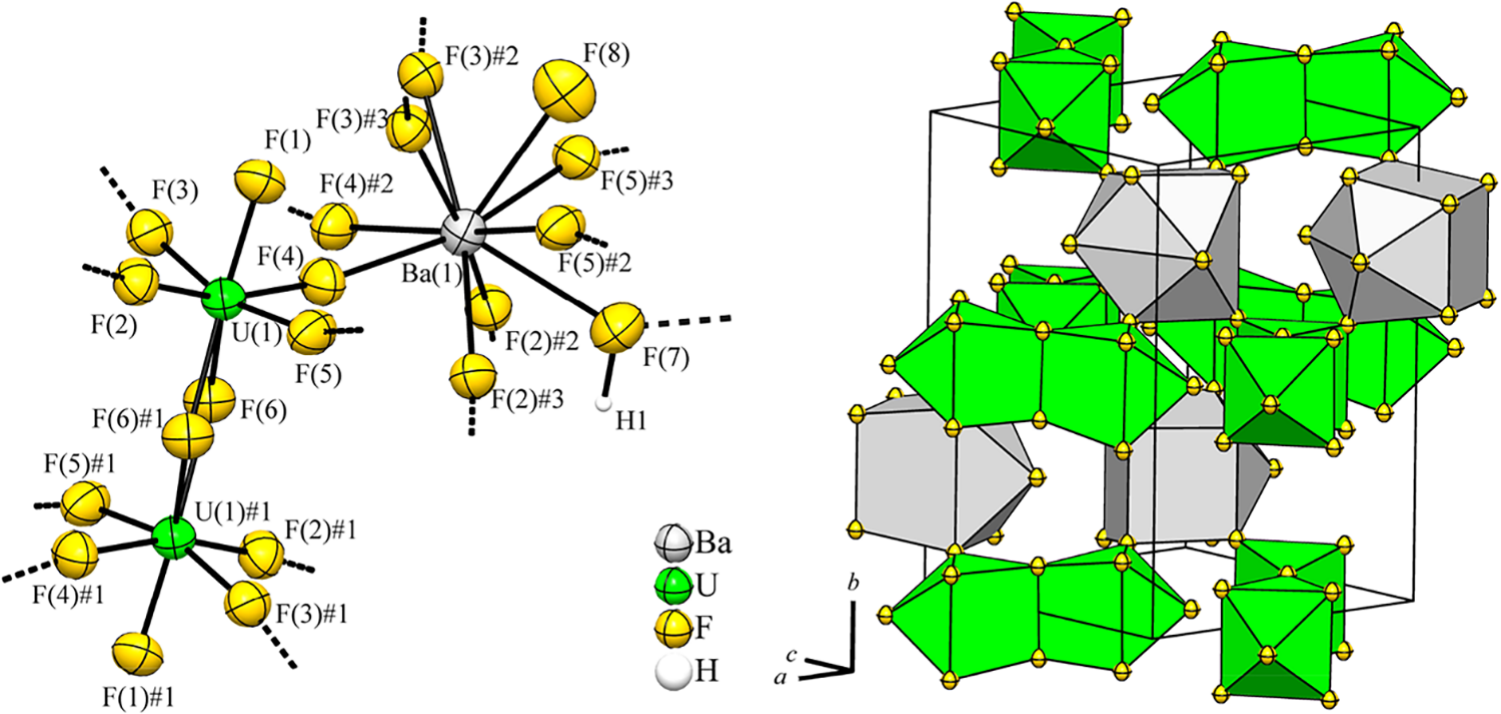
Section of the crystal structure of Ba­[U_2_F_12_]·1.36HF on the left with displacement ellipsoids at 70% probability at 100 K, and on the right its crystal structure, with atoms drawn with arbitrary radii. The first coordination spheres of the U atoms are shown as green coordination polyhedra and those of the Ba atoms in gray. Symmetry transformations for the generation of equivalent atoms: U(1)#1 – *x*, 1 – *y*, 1 – *z*; F(1)#1– *x*, 1 – *y*, 1 – *z*; F(2)#1 – *x*, 1 – *y*, 1 – *z*; F(2)#2 1/2 – *x*, 1/2 + *y*, −1/2 + *z*; F(2)#3 1/2 – *x*, 1 – *y*, −1/2 + *z*; F(3)#1 1/2 + *x*, *y*, 3/2 – *z*; F(3)#2 1/2 + *x*, 3/2 – *y*, 3/2 – *z*; F­(3)#3 1/2 + *x*, *y*, 3/2 – *z*; F(4)#1 – *x*, 1 – *y*, 1 – *z*; F(4)#2 *x*, 3/2 – *y*, *z*; F(5)#1 – *x*, 1 – *y*, 1 – *z*; F(5)#2 1 – *x*, 1 – *y*, 1 – *z*; F(5)#3 1 – *x*, 1/2 + *y*, 1 – *z*; F(6)#1 – *x*, 1 – *y*, 1 – *z*. The site occupancy factor of F(8) is only 0.36(4).

The asymmetric unit contains a uranium atom U(1), which is located at the Wyckoff position 8*d* with site symmetry 1.^31^ In the first coordination sphere, the U atom is coordinated by seven fluorine atoms, which are located at Wyckoff position 8*d*. The two F atoms F(6) act μ-bridging between two U(1) atoms, and a dinuclear [U_2_F_12_]^2–^ anion is formed. The U–F bond lengths agree within their tripled standard deviation with those of Sr­[U_2_F_12_],^24^ with the U–F bond lengths of the μ-bridging F atoms are approximately 0.2 Å longer than the other U–F bond lengths.

The equivalent atoms are generated by symmetry transformations; for these, see the caption of Figure 8. The coordination sphere of the dinuclear [U_2_F_12_]^2–^ anion can be described as two monocapped trigonal prisms with a shared edge.

The Ba atom is located at Wyckoff position 4*c* with site symmetry *m*.^31^ It is coordinated by eight fluorine atoms with a smaller distance of approximately 2.70 Å and two atoms with a larger distance of approximately 2.91 Å. The latter fluorine atoms F(7) and F(8) belong to HF molecules of crystallization, as can be seen in Figure 8. The site occupancy factor of F(8) is only 0.36(4), and therefore, the respective H atom could not be located. It is likely that the compound Ba­[U_2_F_12_]·2HF exists, but during the removal of the solvent in vacuum, a part of the HF molecules of crystallization seem to be removed, too. The shape of the coordination polyhedron can be described as a distorted bifold-capped cube. In the homologue compound Sr­[U_2_F_12_], the coordination number of the Sr^2+^ cation is eight with Sr–F distances of 2.479(4) Å.

Overall, a 3D network is formed by the corner shared coordination polyhedra of the barium and uranium atoms.

### Structural-Chemical Classification of Alkali Metal Hexafluoridouranates­(V)

The structural-chemical properties of alkali metal hexafluoridouranates­(V) shall be compared with those of other representatives of the composition *M*[*E* ^V^F_6_] (*M* = Li–Cs; *E* ^V^ = P–Bi, Pa, Np, Pu). Previous work is available for hexafluoridopnictates­(V)^70^ and various hexafluorometallate­(V) salts.^71^ Structural data for the actinoids, with the exception of uranium, are sparsely available for this class of compounds so that they can only be included in some places. Table 9 lists structural parameters such as structure type, space group, lattice parameters, and the literature reference of the compounds under consideration. Data from high-pressure modifications were not listed (NaSbF_6_ (rhombohedral), KPF_6_ (cubic)).^72,73^ The lithium salts with hexafluoridopnictate­(V) and uranate­(V) ions all crystallize in the trigonal crystal system, isotypic to LiSbF_6_, in space group *R*3̅ (No. 148).^74^ The coordination number of the cations is six.

**Table 9:** Overview of Crystal Structures of Compounds with Composition *M*[*E* ^V^F_6_] (*M* = Li, Na, K, Rb, and Cs; *E* ^V^ = P, As, Sb, Bi, Pa, U, Np, and Pu)

*M*	*E* ^V^	structure type	space group (No.)	*a*/Å	*b*/Å	*c*/Å	*V*/Å^3^	Lit.
Li	P	LiSbF_6_	*R*3̅ (148)^a^	4.932	*a*	12.658	266.65	^44^
	As			5.016	*a*	13.028	283.87	^44^
	Sb			5.18	*a*	13.60	316.03	^9^
	Bi			5.181	*a*	13.99	325.22	^49^
	U			5.1902(7)	*a*	14.265(3)	332.78(1)	this work
Na	P	NaSbF_6_	*Fm*3̅*m* (225)	7.6140	*a*	*a*	441.41	^51^
	As	LiSbF_6_	*R*3̅ (148)^a^	5.3375	*a*	13.9645	344.53	^77^
	As	NaSbF_6_	*Fm*3̅*m* (225)	7.8608	*a*	*a*	485.74	^77^
	Sb	NaSbF_6_	*Fm*3̅*m* (225)	8.203	*a*	*a*	551.97	^78^
	Bi	LiSbF_6_	*R*3̅ (148)^a^	5.468	*a*	15.16	392.54	^49^
	U	LiSbF_6_	*R*3̅ (148)^a^	5.4101(8)	*a*	15.746(3)	399.12(1)	this work
	U	NaSbF_6_	*Fm*3̅*m* (225)	8.608	*a*	*a*	637.83	^17^
	Pa	?	?	5.35	*a*	3.98	113.92	^15^
K	P	KPF_6_	*Fm*3̅*m* (225)	7.7891	*a*	*a*	472.57	^51^
	As	KAsF_6_	*R*3̅ (148)^a^	7.348	*a*	7.274	340.13	^57^
	Sb	KNbF_6_	*P*4̅2*m* (111)	5.16	*a*	10.07	268.12	^44,59^
	Sb	KSbF_6_	*Ia*3̅ (206)	10.15	*a*	*a*	1045.68	^60^
	Bi	KNbF_6_	*P*4̅*c*2 (116)	5.248	*a*	10.07	277.34	^55^
	Bi	KSbF_6_	*Ia*3̅ (206)	10.34	*a*	*a*	1105.51	^55^
	U	KUF_6_	*C*2/*m* (12)	11.4415(2)	8.0345(1)	5.5655(1)	511.62(4)	this work
	Pa	?	?	5.64	11.54	7.98	519.38	^15^
Rb	P	KPF_6_	*Fm*3̅*m* (225)	7.887	*a*	*a*	490.61	^51^
	As	KAsF_6_	*R*3̅ (148)^a^	7.497	*a*	7.589	369.39	^61^
	As	CsPF_6_	*Fm*3̅*m* (225)	8.246	*a*	*a*	560.7	^61^
	Sb	KAsF_6_	*R*3̅ (148)^a^	7.670	*a*	7.861	400.50	^61^
	Sb	Ca□B_6_	*Pm*3̅*m* (221)	5.287	*a*	*a*	147.8	^61^
	Bi	KAsF_6_	*R*3̅ (148)^a^	7.712	*a*	7.889	406.34	^49^
	U	KUF_6_	*C*2/*m* (12)	11.797(2)	8.0167(2)	5.7272(1)	541.64(2)	this work
	Pa	RbPaF_6_	*Cmme* (67)	8.0483	12.0253	5.8608	567.23	^12^
Cs	P	KPF_6_	*Fm*3̅*m* (225)	8.197	*a*	*a*	550.76	^51^
	As	KAsF_6_	*R*3̅ (148)^a^	7.723	*a*	8.050	415.81	^61^
	As	CsPF_6_	*Fm*3̅*m* (225)	8.384	*a*	*a*	589.32	^61^
	Sb	KAsF_6_	*R*3̅ (148)^a^	7.904	*a*	8.261	446.95	^62^
	Sb	Ca□B_6_	*Pm*3̅*m* (221)	5.474	*a*	*a*	164.03	^61^
	Bi	KAsF_6_	*R*3̅ (148)^a^	7.930	*a*	8.274	450.60	^49^
	U	KAsF_6_	*R*3̅ (148)^a^	8.019	*a*	8.437	469.9	^64^
	Pa	?	?	6.14	12.56	8.06	621.57	^15^
	Np	KAsF_6_	*R*3̅ (148)^a^	8.017	*a*	8.386	466.78	^79^
	Pu	?	?	8.006	*a*	8.370	464.61	^80^

a Hexagonal setting.

A partial transition to the cubic crystal system is already observed for the sodium salts. Except for the P, Bi, and Pa containing salts, polymorphism was observed in the sodium salts. In addition to the rhombohedral LiSbF_6_ type, the cubic NaSbF_6_ type (C.N­(*M*) = 6), space group *Fm*3̅*m* (No. 225), occurs.^72^ The latter is related to the NaCl type.^70,75^ The structural relationship between the rhombohedral and cubic phases can be shown with the help of a group–subgroup relationship in the form of a Bärnighausen family tree, see Figure 9.^41,63^

**Figure 9:**
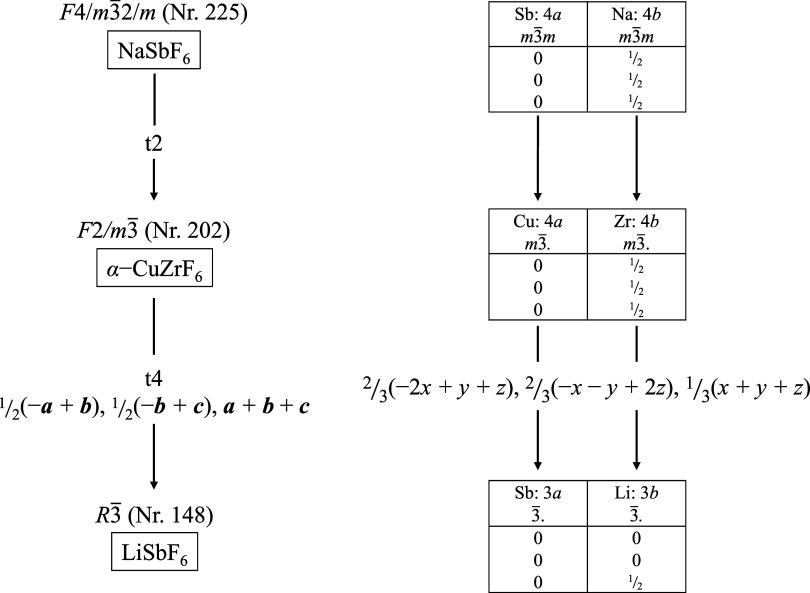
Bärnighausen tree with group–subgroup relations of aristotype NaSbF_6_ via α–CuZrF_6_ to hettotype LiSbF_6_.

Starting from the aristotype NaSbF_6_, space group *F*4/*m*3̅2/*m* (No. 225), a *translationengleiche* transition of index 2 leads to space group *F*2/*m*3̅ (No. 202) with the hettotype α-CuZrF_6_.^26,65,76^ During the transition, the point symmetry is decreased from *O_h_* to *T_h_* symmetry. Further symmetry reduction with a *translationengleiche* transition of index 4, under transformation of coordinates and axes into the hexagonal setting, leads to subgroup *R*3̅ (No. 148) with the hettotype LiSbF_6_. The lower site symmetry of 3̅ (*S*_6_ symmetry) is then obtained for the positions.

Compared to the lighter homologues, the potassium salts show more diversity in the crystal systems and thus structure types, which can be attributed to the accessibility of different coordination numbers for the K^+^ ions. In addition to the cubic crystallizing representatives, with PF_6_^–^, SbF_6_^–^, or BiF_6_^–^ ions, tetragonal ones are also observed, which are isotypic to KNbF_6_ (C.N.(*M*): 8) or a distortion variant thereof.^70^ However, the uranium compound shows the greatest structural difference in the series. In addition to the monoclinic crystal system, instead of molecular UF_6_^–^ ions, one-dimensional infinite chains of the form _∞_^1^{[UF_4/1_F_4/2_]^−^} are observed. In KPaF_6_, bridged anions are likely also formed, which can be assumed based on the lattice parameters similar to RbPaF_6_; single-crystal structure data are not available.^38–40^ The coordination number of the K^+^ cation in KUF_6_ is 10.

The transition to rubidium salts shows no major anomalies for the hexafluoridopnictates­(V). Cubic and rhombohedral variants are observed, which often crystallize in the KAsF_6_ structure type.^81^ The UF_6_^–^ and PaF_6_^–^ salts also show with bridged _∞_^1^{[*E*F_4/1_F_4/2_]^−^} ions a different behavior. There is a modification of RbSbF_6_ that crystallizes in the CaB_6_ structure type, which is also related to the CsCl type and can be written as Ca□B_6_ type (C.N.(*M*): 6, □: unoccupied octahedral void).^70,82^ Of the latter, a relationship with the KAsF_6_ structure type (C.N.(*M*): 12) can be shown via a group–subgroup relationship, see Figure 10. From the cubic aristotype Ca□B_6_, space group *P*4/*m*3̅2/*m* (No. 221), in which the octahedral voids on the 1*b* position are unoccupied, a *translationengleiche* transition of index 4 leads to subgroup *R*3̅2/*m* (No. 166). In the hettotype BaSiF_6_ the 1*b* position is occupied by Ba^2+^ cations.^83^ Further symmetry reduction by a *translationengleiche* transition of index 2 leads to subgroup *R*3̅ (No. 148), in which KAsF_6_ is represented.

**Figure 10:**
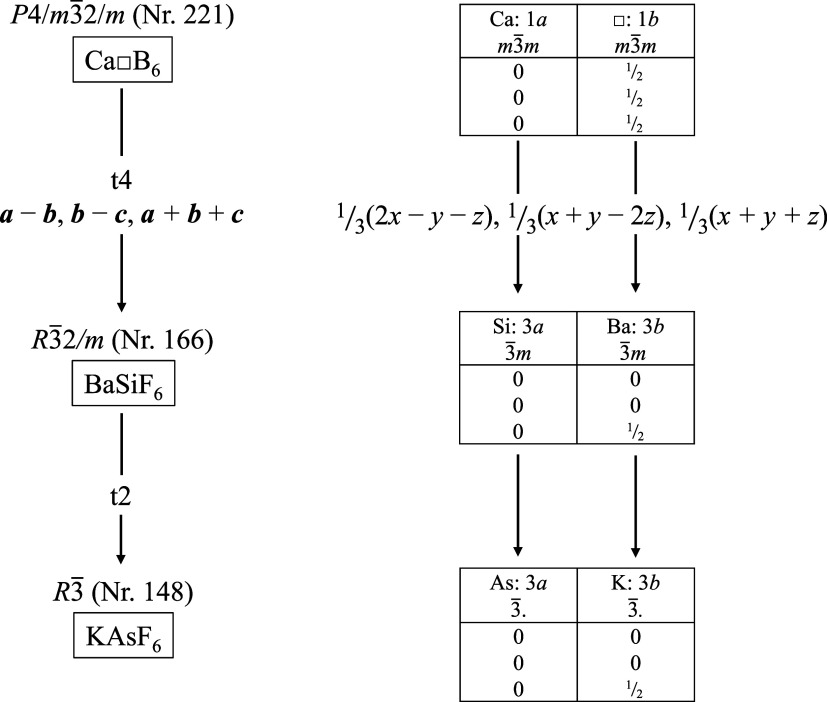
Bärnighausen tree with group–subgroup relation of the aristotype Ca□B_6_ via BaSiF_6_ to the hettotype KAsF_6_.

The cesium salts show differences in relation to the other alkali metal actinoid compounds. The fluoridouranate, -neptunate, and -plutonate crystallize in the rhombohedral crystal system with molecular *E*F_6_^–^ ions, while the protactinate likely crystallizes isotypic to RbPaF_6_ in the orthorhombic system with infinite strands of anions.^40,84^ This raises the question of the driving force behind the interconnection of the anions with respect to the electron configuration. On the one hand, the diamagnetic Pa­(V) compounds ([Rn]­5f^0^) show bridged anions from potassium to cesium, which, however, cannot be attributed to a Pa···Pa interaction. On the other hand, the paramagnetic U­(V) compounds ([Rn]­5f^1^) show bridged anions in the potassium and rubidium salt and molecular anions in the cesium salt.^27^ The cesium hexafluoridopnictates­(V) crystallize, just like the lighter homologues, in either the cubic or trigonal crystal system.

Finally, the relationships and data shown in Table 9 are summed up graphically in a structure field diagram in Figure 11, see also the literature.^70^ The observed structure types of the corresponding *ME*(V)­F_6_ compounds are plotted as a function of the effective ionic radii of the cation.^36^ The diagram is divided into three colored areas, which are intended to show the relationship to the aristotypes, i.e., the NaCl and CsCl, or the KUF_6_ structure type. Table 10 lists selected crystallographic data, such as the Wyckoff position of the alkali metal ion *M* or the coordination number of *M* of the observed structure types.

**Figure 11:**
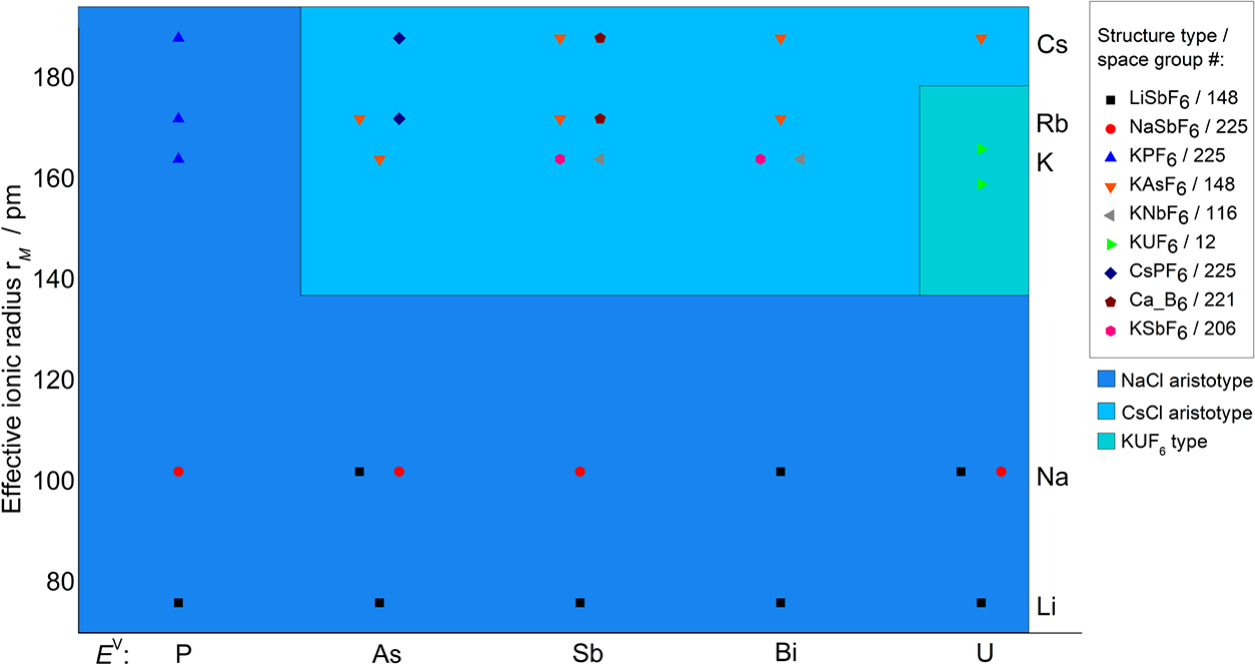
Structure field diagram of fluorides with composition *ME*(V)­F_6_ (*M* = Li, Na, K, Rb, and Cs; *E* ^V^ = P, As, Sb, Bi, and U), with color-coded areas to show the structural relationships. The effective ionic radii of the cations *r*_*M*_ were chosen in accordance with the coordination number; those of the *E*(V)-Ions are for C.N. 6.

**Table 10:** Summary of Crystal-Chemical Data for the Observed Structure Types

structure type	crystal system	Wyckoff position (*M*)	site symmetry	C.N.(*M*):
NaSbF_6_^[127]^	cubic	4*b*	3̅.	6
KSbF_6_^[134]^	cubic	8*b*	.3̅.	6
KPF_6_^[128]^	cubic	4*b*	*m*3̅*m*	-^a^
CsPF_6_^[137]^	cubic	4*a*	*m*3̅*m*	-^a^
Ca□B_6_^[138]^	cubic	1*b*	*m*3̅*m*	6
KAsF_6_^[130]^	trigonal	3*b*	3̅.	12
LiSbF_6_^[89]^	trigonal	3*b*	3̅.	6
KNbF_6_^[132]^	tetragonal	2*c*	4̅..	8
KUF_6_ ^b^	monoclinic	4*h*	2	10

a Disordered structures.

b Single-crystal studies of this work.

## Conclusions

We presented the synthesis of the compounds *M*UF_6_ (*M* = Li–Cs, Tl) by the reaction of β-uranium­(V) fluoride with the respective fluoride *M*F in aHF. This route also allowed us to obtain these compounds in the form of single crystals. The oxonium salt H_3_OUF_6_ was obtained by the reaction of β-UF_5_ with silica glass wool in aHF. Other forms of silicon dioxide, such as amorphous or annealed SiO_2_, led to the formation of unidentified solid side products. AgUF_6_ was synthesized in aHF from Ag and UF_6_ in the presence of CO.

The salts LiUF_6_ and NaUF_6_ (rhombohedral) crystallize in the trigonal crystal system and are isotypic to LiSbF_6_ with molecular UF_6_^–^ ions in the solid state. The crystal structures are related to the NaSbF_6_ structure type, which presents a hettotype of the NaCl structure type. This was shown by a group–subgroup relation.

H_3_OUF_6_ crystallizes in the cubic crystal system and is an isotypic KSbF_6_. Molecular UF_6_^–^ ions are present in the solid. Previous studies had not been able to discriminate between a cubic primitive or body-centered Bravais lattice, as the indexing of the reflections in the powder X-ray pattern does not allow for discrimination.

KUF_6_, RbUF_6_, and TlUF_6_ crystallize in the monoclinic crystal system and contain more complex structural motives compared with the compounds mentioned above. In the solids, the anions are one-dimensional (1D) infinite strands of the type _∞_^1^{[UF_4/1_F_4/2_]^−^}. The U atoms have a coordination number of 8 and the coordination polyhedron is a distorted trigonal dodecahedron. There was uncertainty in the literature regarding the anions present and the crystal system of these salts. Various orthorhombic space groups were assigned to the compounds, but these could not be confirmed in this work. On the one hand, no structural model from the single-crystal structure investigations could be satisfactorily refined in orthorhombic space groups and, on the other hand, the powder X-ray diffractograms showed reflection splitting, which indicated the monoclinic crystal system. However, the data described in the literature could also have been obtained on other modifications of the compounds, but no evidence for such polymorphism could be found in this work.

A coordination of the U atoms similar to that in KUF_6_, RbUF_6_, and TlUF_6_ was observed in AgUF_6_. By single-crystal X-ray diffraction, we could establish the space group to be *P*4_2_/*m*. Attempts to determine and refine the structure in the previously proposed space group *P*4_2_/*mcm* resulted in a completely inappropriate structure model. AgUF_6_ crystallizes in the CaTbF_6_ structure type. The coordination sphere of the Ag^+^ cations is octahedron-like or distorted pentagonal antiprismatic if longer Ag–F distances are considered. The alkali metal cations in KUF_6_ and RbUF_6_ show a distorted pentagonal-prismatic coordination sphere.

The dodecafluoridodiuranate­(V) Ba­[U_2_F_12_]·1.36HF was synthesized, and its crystal structure was determined. In contrast to the only other known compound containing the [U_2_F_12_]^2–^ dianion, Sr­[U_2_F_12_], the barium compound contains HF molecules of crystallization.

## Experimental Section

### General

All operations were performed on a Monel steel Schlenk line, which was passivated with fluorine at various temperatures and pressures before use. Reaction vessels were made out of fluoropolymer (perfluoroalkoxy alkanes, PFA) and sealed with a suitable needle valve (Swagelok). The vessels were baked out in vacuum (∼10^–3^ mbar) at ca. 100 °C several times and passivated with diluted F_2_ (F_2_/Ar 20:80, v/v, Solvay). Alkali metal and alkaline earth metal fluorides had been purchased from Merck and purified according to literature methods.^85^ Solid starting materials were stored and handled in an Ar-filled (Ar 5.0, Nippon Gases) glovebox (MBraun).

**Caution!** HF is highly toxic. Therefore, proper protective equipment must be worn, and appropriate emergency treatment procedures must be available in the case of contact. Uranium compounds are radioactive, and appropriate or required measures for their handling need to be taken.

The preparation of β-UF_5_ was carried out by the photoreduction of UF_6_ with CO in a UV reactor.^7,86^ The purity of the obtained β-uranium­(V) fluoride was analyzed by powder X-ray diffraction and IR spectroscopy; see the Supporting Information, Figures S11 and S12.

To prepare the alkali metal hexafluoridouranates­(V), one FEP Schlenk tube each was dried at 150 °C in a drying oven and transferred to the glovebox. There it was charged with equimolar quantities of alkali metal fluoride and β-uranium­(V) fluoride, attached to a Schlenk line outside of the glovebox, and then aHF was distilled onto the solids in a vacuum under cooling with liquid nitrogen. Exemplary quantities used are listed in Table S1. The reaction vessel was heated in an air bath until the hydrogen fluoride melted; as soon as it became a liquid, the majority of the solids dissolved, turning the solution blue. At room temperature, the reaction mixtures were homogenized by vortexing, and after a short time, the solvent was distilled into a separate FEP Schlenk tube at a low flow rate under vacuum. This led to the formation of crystalline products, photographs of which are shown in Figures S1–S3. Several vacuum inert gas purges were carried out to remove traces of the solvent. Typical batch sizes for the syntheses of the various hexafluoridouranates­(V) are given in the Supporting Information, Table S1.

AgUF_6_ was obtained as a side product from the reaction of Ag powder with UF_6_ in aHF under a CO atmosphere. A PFA-vessel with an approximate volume of 4 mL was loaded with Ag powder (103.6 mg, 0.96 mmol) and closed with a PFA valve. At −196 °C, aHF (1 mL) and UF_6_ (880.0 mg, 2.50 mmol) were condensed into the vessel, and CO (2 bar) was added. The reaction was warmed to −40 °C, and a colorless solution and a colorless solid were observed. For several times, all volatile compounds were pumped off at −196 °C and fresh CO was added. After 4 weeks, the reaction vessel was warmed to room temperature, and all volatile compounds were removed in static vacuum. The obtained colorless solid (254.0 mg, 57% with respect to the used Ag) was dried for only a short time in dynamic vacuum, to prevent a potential carbonyl compound from decomposition. A few colorless needle-like crystals were found beneath greater agglomerates of yellowish solid, which were identified as AgUF_6_. Unfortunately, no powder X-ray diffraction pattern could be obtained.

For the synthesis of Ba­[U_2_F_12_]·1.36HF, an FEP Schlenk tube was charged with BaF_2_ (26.3 mg, 0.15 mmol) and UF_5_ (50 mg, 0.15 mmol) and 2 mL of aHF was condensed onto the solids in vacuum under cooling with liquid nitrogen. The vessel was heated to 80 °C for 2 h, and the solution changed its color to blue. Subsequent storage at −40 °C yielded greenish plate-like crystals. The solvent was removed under static vacuum at room temperature, and Ba­[U_2_F_12_]·1.36HF was isolated (isolated yield: 35.8 mg, 0.04 mmol, 27% with respect to BaF_2_). Unfortunately, no powder X-ray pattern could be obtained.

CCDC 2468468 (LiUF_6_), 2468469 (NaUF_6_), 2468470 (AgUF_6_), 2468471 (RbUF_6_), 2468472 (TlUF_6_), 2468473 (KUF_6_), 2468474 (H_3_OUF_6_), and 2468676 (Ba­[U_2_F_12_]·1.36HF) contain the supplementary crystallographic data for this paper. These data are provided free of charge by The Cambridge Crystallographic Data Centre.

## Supplementary Material



## Notes

Typical batch sizes of the syntheses, photographs of most compounds, selected crystallographic data of the structure determinations, powder X-ray diffraction patterns of most bulk phase syntheses, and the characterization of the UF_5_ used as a starting material (PDF)
