# Supplementary material for: Syntheses, Single-Crystal Structures, and Structural Chemistry of Hexafluoridouranates(V), MUF6 (M = Li–Cs, Ag, Tl, H3O), and the Dodecafluoridodiuranate(V) Ba[U2F12]·1.36HF
Source: Inorg Chem. 2025 Sep 4;64(36):18432–46. doi: 10.1021/acs.inorgchem.5c03005 (PMC12442095; doi:10.1021/acs.inorgchem.5c03005)
Supplement: Supplementary file 1 [file ic5c03005_si_001.pdf]

Supporting Information

Syntheses, Single Crystal Structures, and Structural-Chemistry of Hexafluoridouranates(V), $\mathit{M}UF_6$ (M = Li−Cs, Ag, Tl, H_3O) and the Dodecafluoridodiuranate(V) $Ba[U_2F_{12}]\cdot 1.36HF$

Benjamin Scheibe,[a] Tobias B. Wassermann,[a] Florian Kraus[a*]

[a] Dr. Benjamin Scheibe, M. Sc. Tobias B. Wassermann, Prof. Dr. Florian Kraus, Arbeitsgruppe Fluorchemie, Anorganische Chemie, Fachbereich Chemie, Philipps-Universität Marburg, Hans-Meerwein-Str. 4, 35032 Marburg, Germany, f.kraus@uni-marburg.de, https://www.uni-marburg.de/de/fb15/arbeitsgruppen/anorganische_chemie/ag-kraus

Content

Typical batch sizes for the syntheses of hexafluoridouranates(V) 3

Photographs of the various hexafluoridouranates(V) 4

Selected crystallographic data, details of the structure determinations, atomic coordinates, and displacement parameters of the hexafluoridouranates(V) 5

$LiUF_6$....................5

$NaUF_6$ – rhombic polymorph....................7

KUF_6 9

$RbUF_6$ 11

$AgUF_6$ 13

$TlUF_6$....................15

H_3OUF_6 17

$Ba[U_2F_{12}]\cdot HF$ 19

Powder X-ray diffraction patterns of the hexafluoridouranates(V) 21

$LiUF_6$....................21

$NaUF_6$ – rhombic and cubic polymorphs....................22

KUF_6 23

$RbUF_6$ 24

$CsUF_6$ 25

$TlUF_6$....................26

H_3OUF_6 27

Characterization of UF_5 used as a starting material....................28

Literature....................30

Typical batch sizes for the syntheses of hexafluoridouranates(V)

Table S1: Exemplary quantities of fluorides *M*F and β-uranium(V) fluoride used for the preparation of hexafluoridouranates(V) in anhydrous hydrogen fluoride as well as the isolated yield. The percentage yield refers to the amount of β-UF_5 used.

M:	*M*F		*β*–UF_5		V_{aHF} / mL	Yield	
	m / mg	*n* / µmol	*m* / mg	*n* / µmol		*m* / mg	%
Li	19.80	763	254.3	764	10	226	82
Na	15.84	377	125.5	377	4	97.8	69
K	43.83	754	251.2	754	4	237	80
Rb	79.04	757	252.0	757	4	276	83
Cs	114.3	752	250.8	753	2	295	81
Tl	71.80	321	107.2	322	5	107.8	60
NH_4^+	24.96	674	224.7	675	3	215.0	86

Photographs of the various hexafluoridouranates(V)

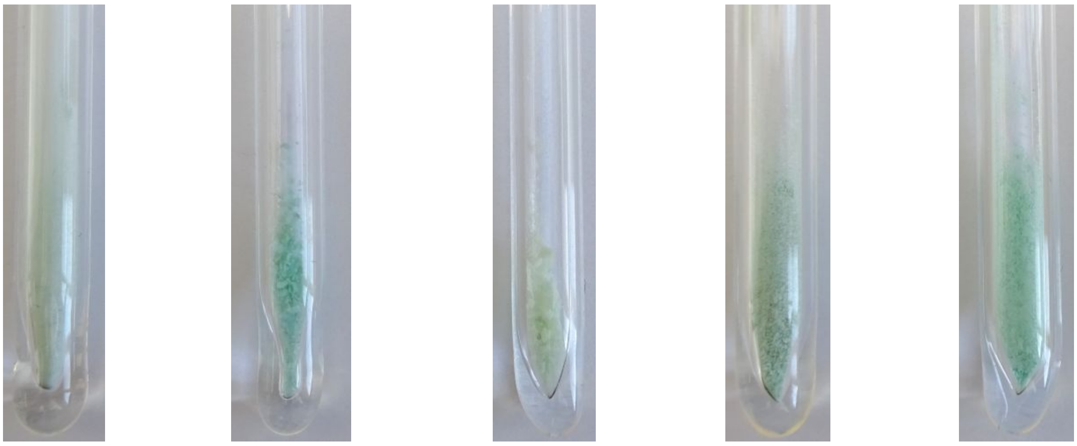

$LiUF_6$ $NaUF_6$ KUF_6 $RbUF_6$ $CsUF_6$

Figure S1: Photographs of the obtained alkali metal hexafluoridouranates(V) in flame-sealed silica glass ampules under argon atmosphere.

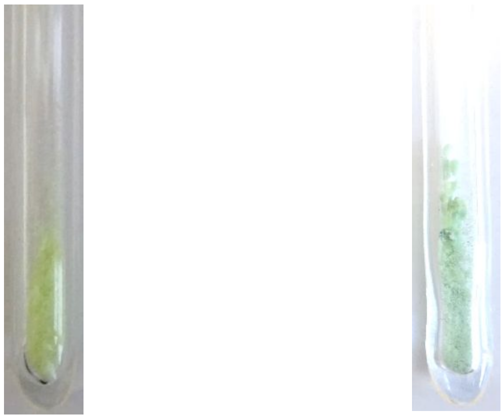

$TlUF_6$ NH_4UF_6

Figure S2: Photographs of the obtained thallium(I) hexafluoridouranate(V) and ammonium hexafluoridouranate(V) in flame-sealed silica glass ampules under argon atmosphere.

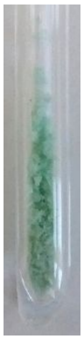

Figure S 3: Photograph of the obtained oxonium hexafluoridouranate(V) in flame-sealed silica glass ampules under argon atmosphere.

Selected crystallographic data, details of the structure determinations, atomic coordinates, and displacement parameters of the hexafluoridouranates(V)

$LiUF_6$

Table S2: Selected crystallographic data and details of the structure determination of $LiUF_6$.

	$LiUF_6$ (SCXRD)
Diffractometer	STOE IPDS 2
Empirical formula	F_6LiU
Color and habitus	blueish plate
Crystal size / mm^3	0.16 · 0.12 · 0.08
Molar Mass / g·mol^{-1}	358.97
Crystal system	trigonal
Space group (No.)	$R\bar{3}$ (148)
a^{hex} / Å	5.1902(7)
c^{hex} / Å	14.265(3)
V^{hex} / Å^3	332.78(1)
Z	3
$\rho_{ber.}$ / g·cm^{-3}	5.374
λ / Å	0.71073 (Mo-K$_\alpha$)
T / K	100
μ / mm^{-1}	36.61 (Mo-K$_\alpha$)
θ_{max}	31.94
hkl_{max}	$-3 \leq h \leq 7$
	$-7 \leq k \leq 5$
	$-21 \leq l \leq 16$
R_{int}, R_σ	0.029, 0.022
$R(F)$ ($I \geq 2\sigma(I)$, all data)	0.014, 0.014
$wR(F^2)$ ($I \geq 2\sigma(I)$, all data)	0.030, 0.030
S (all data)	1.12
Data, parameters, restraints	268, 15, 0
$\Delta\rho_{max}$, $\Delta\rho_{min}$ / e·Å^{-3}	1.48, −1.78

Table S3: Atomic coordinates, Wyckoff positions, site symmetries, and isotropic displacement parameters U_{eq} of $LiUF_6$.

Atom	Wyckoff position	Site symmetry	x	y	z	U_{eq} / Å^2
U(1)	3*a*	$\bar{3}$.	2/3	1/3	1/3	0.00556(12)
Li(1)	3*b*	$\bar{3}$.	1/3	2/3	1/6	0.011(2)
F(1)	18*f*	1	0.3628(4)	0.3702(4)	0.24956(12)	0.0100(3)

Table S4: Anisotropic displacement parameters U^{ij} of $LiUF_6$ at 100 K.

Atom	U^{11} / Å^2	U^{22} / Å^2	U^{33} / Å^2	U^{23} / Å^2	U^{13} / Å^2	U^{12} / Å^2
U(1)	0.00616(13)	0.00616(13)	0.00435(14)	0	0	0.00308(6)
Li(1)	0.012(3)	0.012(3)	0.011(5)	0	0	0.0058(16)
F(1)	0.0103(7)	0.0112(7)	0.0091(6)	0.0007(6)	−0.0015(6)	0.0059(6)

$NaUF_6$ – rhombic polymorph

Table S5: Selected crystallographic data and details of the structure determination of $NaUF_6$, rhombic polymorph.

	$NaUF_6$ (SCXRD)
Diffractometer	STOE IPDS 2T
Empirical formula	F_6NaU
Color and habitus	blueish block
Crystal size / mm^3	0.13 · 0.08 · 0.07
Molar Mass / g·mol^{-1}	375.02
Crystal system	trigonal
Space group (No.)	$R\bar{3}$ (148)
a^{hex} / Å	5.4101(8)
c^{hex} / Å	15.746(3)
V^{hex} / Å^3	399.12(1)
Z	3
$\rho_{ber.}$ / g·cm^{-3}	4.68
λ / Å	0.71073 (Mo-K_α)
T / K	100
μ / mm^{-1}	30.61 (Mo-K_α)
θ_{max}	27.75
hkl_{max}	$-7 \leq h \leq 7$
	$-6 \leq k \leq 6$
	$-18 \leq l \leq 20$
R_{int}, R_σ	0.115, 0.062
$R(F)$ ($I \geq 2\sigma(I)$, all data)	0.029, 0.029
$wR(F^2)$ ($I \geq 2\sigma(I)$, all data)	0.074, 0.074
S (all data)	1.11
Data, parameters, restraints	212, 12, 0
$\Delta\rho_{max}$, $\Delta\rho_{min}$ / e·Å^{-3}	2.03, −1.20

Table S6: Atomic coordinates, Wyckoff positions, site symmetries, and isotropic displacement parameters U_{eq} of $NaUF_6$ (rhombohedral polymorph).

Atom	Wyckoff position	Site symmetry	x	y	z	U_{eq} / Å^2
U(1)	3a	$\bar{3}$.	2/3	1/3	1/3	0.0132(3)
Na(1)	3b	$\bar{3}$.	1/3	2/3	1/6	0.0146(16)
F(1)	18f	1	0.6429(1)	0.6314(1)	0.2570(3)	0.0191(11)

Table S7: Anisotropic displacement parameters U^{ij} of $NaUF_6$ (rhombohedral polymorph) at 100 K.

Atom	U^{11} / Å²	U^{22} / Å²	U^{33} / Å²	U^{23} / Å²	U^{13} / Å²	U^{12} / Å²
U(1)	0.0119(3)	0.0119(3)	0.0159(4)	0	0	0.00593(16)
Na(1)	0.013(2)	0.013(2)	0.018(4)	0	0	0.0064(12)
F(1)	0.022(3)	0.017(3)	0.021(2)	0.002(2)	0.000(2)	0.012(2)

KUF_6

Table S8: Selected crystallographic data and details of the structure determination of KUF_6.

	KUF_6 (SCXRD)
Diffractometer	Bruker D8 Quest
Empirical formula	F_6KU
Color and habitus	blueish needle
Crystal size / mm^3	0.42 · 0.09 · 0.08
Molar Mass / g·mol^{-1}	391.13
Crystal system	monoclinic
Space group (No.)	*C*2/*m* (12)
a / Å	11.442(2)
b / Å	8.0345(10)
c / Å	5.5655(10)
β / °	90.138(9)
V / Å^3	511.62(4)
Z	4
$\rho_{ber.}$ / g·cm^{-3}	5.08
λ / Å	0.71073 (Mo-K$_\alpha$)
T / K	100
μ / mm^{-1}	32.57 (Mo-K$_\alpha$)
θ_{max}	36.31
hkl_{max}	$-19 \le h \le 19$
	$-13 \le k \le 13$
	$-9 \le l \le 9$
R_{int}, R_σ	0.019, 0.006
$R(F)$ ($I \ge 2\sigma(I)$, all data)	0.027, 0.028
$wR(F^2)$ ($I \ge 2\sigma(I)$, all data)	0.074, 0.076
S (all data)	1.14
Data, parameters, restraints	1303, 43, 0
$\Delta\rho_{max}$, $\Delta\rho_{min}$ / e·Å^{-3}	5.61, −5.62

Table S9: Atomic coordinates, Wyckoff positions, site symmetries, and isotropic displacement parameters U_{eq} of KUF_6.

Atom	Wyckoff position	Site symmetry	*x*	*y*	*z*	U_{eq} / Å²
U(1)	4*h*	2	1/2	0.75044(2)	1/2	0.00466(10)
K(1)	4*i*	*m*	0.7526(1)	1	0.9642(3)	0.0161(2)
F(1)	8*j*	1	0.3985(2)	0.8027(4)	0.7965(5)	0.0164(5)
F(2)	8*j*	1	0.3539(2)	0.6960(4)	0.2966(5)	0.0156(5)
F(3)	4*i*	*m*	0.5861(3)	1	0.6257(7)	0.0146(6)
F(4)	4*i*	*m*	0.5587(3)	1/2	0.3228(7)	0.0156(6)

Table S10: Anisotropic displacement parameters U^{ij} of KUF_6 at 100 K.

Atom	U^{11} / Å²	U^{22} / Å²	U^{33} / Å²	U^{23} / Å²	U^{13} / Å²	U^{12} / Å²
U(1)	0.00499(12)	0.00355(12)	0.00545(12)	0	0.00035(6)	0
K(1)	0.0125(5)	0.0119(5)	0.0238(5)	0	−0.0011(4)	0
F(1)	0.0149(11)	0.0185(13)	0.0159(11)	−0.0025(10)	0.0026(9)	0.0022(10)
F(2)	0.0140(10)	0.0141(12)	0.0185(11)	−0.0021(9)	−0.0049(9)	−0.0032(9)
F(3)	0.0136(14)	0.0087(13)	0.0215(16)	0	−0.0052(12)	0
F(4)	0.0171(15)	0.0097(14)	0.0201(16)	0	0.0058(13)	0

$RbUF_6$

Table S11: Selected crystallographic data and details of the structure determination of $RbUF_6$.

	$RbUF_6$ (SCXRD)
Diffractometer	STOE IPDS 2T
Empirical formula	F_6RbU
Color and habitus	blue-green block
Crystal size / mm^3	0.07 · 0.05 · 0.04
Molar Mass / g·mol^{-1}	437.50
Crystal system	monoclinic
Space group (No.)	$C2/m$ (12)
a / Å	11.797(2)
b / Å	8.0167(2)
c / Å	5.7272(1)
β / °	90.00(3)
V / Å^3	541.64(2)
Z	4
$\rho_{ber.}$ / g·cm^{-3}	5.37
λ / Å	0.71073 (Mo-K$_\alpha$)
T / K	100
μ / mm^{-1}	38.93 (Mo-K$_\alpha$)
θ_{max}	34.89
hkl_{max}	$-18 \leq h \leq 18$
	$-11 \leq k \leq 12$
	$-9 \leq l \leq 9$
R_{int}, R_σ	0.032, 0.023
$R(F)$ ($I \geq 2\sigma(I)$, all data)	0.028, 0.032
$wR(F^2)$ ($I \geq 2\sigma(I)$, all data)	0.066, 0.068
S (all data)	1.16
Data, parameters, restraints	1243, 43, 0
$\Delta\rho_{max}$, $\Delta\rho_{min}$ / e·Å^{-3}	2.11, −3.14

Table S12: Atomic coordinates, Wyckoff positions, site symmetries, and isotropic displacement parameters U_{eq} of $RbUF_6$.

Atom	Wyckoff position	Site symmetry	*x*	*y*	*z*	U_{eq} / Å²
U(1)	4*h*	2	1/2	0.25040(3)	1/2	0.00964(9)
Rb(1)	4*i*	*m*	0.25294(6)	0	0.04652(12)	0.01359(13)
F(1)	8*j*	1	0.3597(3)	0.1986(5)	0.7015(7)	0.0214(6)
F(2)	8*j*	1	0.6014(3)	0.3008(5)	0.7836(6)	0.0209(6)
F(3)	4*i*	*m*	0.4427(4)	0	0.3311(8)	0.0184(9)
F(4)	4*i*	*m*	0.5804(4)	1/2	0.3744(9)	0.0174(8)

Table S13: Anisotropic displacement parameters U^{ij} of $RbUF_6$ at 100 K.

Atom	U^{11} / Å²	U^{22} / Å²	U^{33} / Å²	U^{23} / Å²	U^{13} / Å²	U^{12} / Å²
U(1)	0.00976(11)	0.00783(12)	0.01134(13)	0	0.00054(6)	0
Rb(1)	0.0129(2)	0.0136(3)	0.0143(3)	0	0.00032(17)	0
F(1)	0.0214(16)	0.0153(15)	0.0274(17)	0.0010(13)	0.0071(12)	−0.0019(13)
F(2)	0.0223(15)	0.0148(15)	0.0256(16)	−0.0010(12)	−0.0059(12)	−0.0014(13)
F(3)	0.024(2)	0.0083(18)	0.023(2)	0	−0.0089(17)	0
F(4)	0.017(2)	0.0079(17)	0.027(2)	0	0.0108(16)	0

$AgUF_6$

Table S14: Selected crystallographic data and details of the structure determination of $AgUF_6$.

	$RbUF_6$ (SCXRD)
Diffractometer	Bruker D8 Venture
Empirical formula	F_6AgU
Color and habitus	light-yellow needle
Crystal size / mm^3	0.123 · 0.068 · 0.032
Molar Mass / g·mol^{-1}	459.90
Crystal system	tetragonal
Space group (No.)	$P4_2/m$ (84)
a / Å	5.4188(2)
c / Å	7.9458(4)
V / Å^3	233.32(2)
Z	2
$\rho_{ber.}$ / g·cm^{-3}	6.546
λ / Å	0.71073 (Mo-K$_\alpha$)
T / K	100
μ / mm^{-1}	38.891(Mo-K$_\alpha$)
θ_{max}	34.89
hkl_{max}	$-9 \leq h \leq 10$
	$-10 \leq k \leq 9$
	$-14 \leq l \leq 15$
Extinction coefficient	0.0017(3)
R_{int}, R_σ	0.0463, 0.008
$R(F)$ ($I \geq 2\sigma(I)$, all data)	0.0115, 0.0139
$wR(F^2)$ ($I \geq 2\sigma(I)$, all data)	0.0266, 0.0277
S (all data)	1.196
Data, parameters, restraints	662, 23, 0
$\Delta\rho_{max}$, $\Delta\rho_{min}$ / e·Å^{-3}	1.363, −0.787

Table S15: Atomic coordinates, Wyckoff positions, site symmetries, and isotropic displacement parameters U_{eq} of $AgUF_6$.

Atom	Wyckoff position	Site symmetry	*x*	*y*	*z*	U_{eq} / Å²
U(1)	2*f*	$\bar{4}$	0	0	1/2	0.00981(5)
Ag(1)	2*a*	2/*m*	1/2	1/2	3/4	0.00492(4)
F(1)	4*j*	*m*	0.31326(19)	0.62450(19)	1	0.00920(18)
F(2)	8*k*	1	−0.19115(14)	0.29439(14)	0.30877(11)	0.01036(14)

Table S16: Anisotropic displacement parameters U^{ij} of $AgUF_6$ at 100 K.

Atom	U^{11} / Å²	U^{22} / Å²	U^{33} / Å²	U^{23} / Å²	U^{13} / Å²	U^{12} / Å²
U(1)	0.00537(4)	0.00537(4)	0.00402(5)	0	0	0
Ag(1)	0.01205(9)	0.00765(8)	0.00973(11)	0	0	0.00346(5)
F(1)	0.0093(4)	0.0125(5)	0.0058(4)	0	0	0.0037(4)
F(2)	0.0099(3)	0.0113(3)	0.0099(3)	0.0009(3)	−0.0012(3)	0.0027(3)

$TlUF_6$

Table S17: Selected crystallographic data and details of the structure determination of $TlUF_6$.

	$TlUF_6$ (SCXRD)
Diffractometer	STOE IPDS 2T
Empirical formula	F_6TlU
Color and habitus	yellow-green needle
Crystal size / mm³	1.2 · 1.1 · 1.0
Molar Mass / g·mol⁻¹	556.40
Crystal system	monoclinic
Space group (No.)	*C*2/*m* (12)
a / Å	11.895(2)
b / Å	7.9983(2)
c / Å	5.6787 (1)
β / °	90.00(3)
V / Å³	540.28(2)
Z	4
$\rho_{ber.}$ / g·cm⁻³	6.84
λ / Å	0.71073 (Mo-K_α)
T / K	100
μ / mm⁻¹	59.75 (Mo-K_α)
θ_{max}	34.89
hkl_{max}	$-19 \leq h \leq 19$
	$-10 \leq k \leq 12$
	$-9 \leq l \leq 9$
R_{int}, R_σ	0.075, 0.055
$R(F)$ ($I \geq 2\sigma(I)$, all data)	0.031, 0.034
$wR(F^2)$ ($I \geq 2\sigma(I)$, all data)	0.081, 0.082
S (all data)	1.14
Data, parameters, restraints	1253, 43, 0
$\Delta\rho_{max}$, $\Delta\rho_{min}$ / e·Å⁻³	3.94, −4.55

Table S18: Atomic coordinates, Wyckoff positions, site symmetries, and isotropic displacement parameters U_{eq} of $TlUF_6$.

Atom	Wyckoff position	Site symmetry	*x*	*y*	*z*	U_{eq} / Å²
U(1)	4*h*	2	1/2	0.74966(3)	1/2	0.00847(11)
Tl(1)	4*i*	*m*	0.24647(2)	1/2	0.95642(6)	0.01253(11)
F(1)	8*j*	1	0.4041(3)	0.6977(4)	0.2083(7)	0.0161(6)
F(2)	8*j*	1	0.6432(3)	0.8036(4)	0.3103(7)	0.0156(6)
F(3)	4*i*	*m*	0.4179(4)	1/2	0.6195(10)	0.0141(9)
F(4)	4*i*	*m*	0.5515(5)	1	0.6785(10)	0.0145(9)

Table S19: Anisotropic displacement parameters U^{ij} of $TlUF_6$ at 100 K.

Atom	U^{11} / Å²	U^{22} / Å²	U^{33} / Å²	U^{23} / Å²	U^{13} / Å²	U^{12} / Å²
U(1)	0.01250(14)	0.00350(14)	0.00940(16)	0	-0.00059(8)	0
Tl(1)	0.01544(15)	0.00964(16)	0.01251(16)	0	-0.00049(9)	0
F(1)	0.0215(15)	0.0092(14)	0.0174(16)	−0.0005(12)	-0.0061(13)	−0.0020(12)
F(2)	0.0188(15)	0.0104(14)	0.0176(16)	0.0019(12)	0.0033(13)	−0.0010(12)
F(3)	0.018(2)	0.0084(18)	0.016(2)	0	0.0039(18)	0
F(4)	0.025(2)	0.0042(16)	0.014(2)	0	-0.0056(19)	0

H_3OUF_6

Table S20: Selected crystallographic data and details of the structure determination of H_3OUF_6.

	H_3OUF_6 (SCXRD)
Diffractometer	STOE IPDS 2
Empirical formula	F_6H_3OU
Color and habitus	blueish block
Crystal size / mm^3	0.11 · 0.10 · 0.10
Molar Mass / g·mol^{-1}	375.02
Crystal system	kubisch
Space group (No.)	$Ia\bar{3}$ (206)
a / Å	10.3504(12)
V / Å^3	1108.8(4)
Z	8
$\rho_{ber.}$ / g·cm^{-3}	4.45
λ / Å	0.71073 (Mo-K$_\alpha$)
T / K	100
μ / mm^{-1}	29.32 (Mo-K$_\alpha$)
θ_{max}	31.98
hkl_{max}	$-15 \leq h \leq 14$
	$-15 \leq k \leq 15$
	$-15 \leq l \leq 15$
R_{int}, R_σ	0.156, 0.046
$R(F)$ ($I \geq 2\sigma(I)$, all data)	0.029, 0.044
$wR(F^2)$ ($I \geq 2\sigma(I)$, all data)	0.063, 0.071
S (all data)	1.17
Data, parameters, restraints	329, 15, 0
$\Delta\rho_{max}$, $\Delta\rho_{min}$ / e·Å^{-3}	1.10, −0.83

Table S21: Atomic coordinates, Wyckoff positions, site symmetries, and isotropic displacement parameters U_{eq} of H_3OUF_6.

Atom	Wyckoff position	Site symmetry	x	y	z	U_{eq} / Å^2
U(1)	8b	.$\bar{3}$.	3/4	1/4	3/4	0.0138(2)
O(1)	8a	.$\bar{3}$.	1/2	0	1/2	0.020(2)
F(1)	48e	1	0.6887(4)	0.0946(4)	0.6428(4)	0.0230(7)

Table S22: Anisotropic displacement parameters U^{ij} of H_3OUF_6 at 100 K.

Atom	U^{11} / Å²	U^{22} / Å²	U^{33} / Å²	U^{23} / Å²	U^{13} / Å²	U^{12} / Å²
U(1)	0.0138(2)	0.0138(2)	0.0138(2)	0.00065(11)	−0.00065(11)	−0.00065(11)
O(1)	0.020(2)	0.020(2)	0.020(2)	−0.006(3)	−0.006(3)	0.006(3)
F(1)	0.025(2)	0.0205(19)	0.0239(19)	−0.0041(15)	−0.0005(15)	0.0010(16)

$Ba[U_2F_{12}]\cdot HF$

Table S23: Selected crystallographic data and details of the structure determination of $Ba[U_2F_{12}]\cdot 1.36HF$.

	$Ba[U_2F_{12}]\cdot 1.36HF$ (SCXRD)
Diffractometer	STOE IPDS 2T
Empirical formula	$BaF_{13.36}H_{1.36}U_2$
Color and habitus	Greenish plate
Crystal size / mm^3	0.18 · 0.16 · 0.10
Molar Mass / g·mol^{-1}	861.41
Crystal system	orthorhombic
Space group (No.)	*Pnma* (62)
a / Å	8.9989(8)
b / Å	12.6822(9)
c / Å	9.3514(6)
V / Å^3	1067.24(14)
Z	4
$\rho_{calc.}$ / g·cm^{-3}	5.404
λ / Å	0.71073 (Mo-K$_\alpha$)
T / K	100
μ / mm^{-1}	34.084 (Mo-K$_\alpha$)
θ_{max}	28.990
hkl_{max}	$-12 \leq h \leq 12$
	$-17 \leq k \leq 17$
	$-11 \leq l \leq 12$
R_{int}, R_σ	0.0253, 0.0134
$R(F)$ ($I \geq 2\sigma(I)$, all data)	0.0366, 0.0447
$wR(F^2)$ ($I \geq 2\sigma(I)$, all data)	0.0916, 0.0949
S (all data)	1.194
Data, parameters, restraints	1481, 85, 0
$\Delta\rho_{max}$, $\Delta\rho_{min}$ / e·Å^{-3}	2.246, −1.874

Table S24: Atomic coordinates, Wyckoff positions, site symmetries, and isotropic displacement parameters U_{eq} of $Ba[U_2F_{12}]\cdot HF$.

Atom	Wyckoff position	Site symmetry	*s.o.f.*	*x*	*y*	*z*	U_{eq} / Å²
Ba(1)	4*c*	*.m.*	1	0.51371(11)	3/4	0.47349(11)	0.0376(2)
U(1)	8*d*	1	1	0.15087(4)	0.51574(4)	0.65427(4)	0.03593(15)
F(1)	8*d*	1	1	0.3218(8)	0.5144(6)	0.8008(8)	0.0419(15)
F(2)	8*d*	1	1	0.0676(7)	0.4023(6)	0.7915(7)	0.0409(14)
F(3)	8*d*	1	1	0.0677(7)	0.6325(5)	0.7851(7)	0.0401(14)
F(4)	8*d*	1	1	0.2853(7)	0.6280(6)	0.5595(7)	0.0432(15)
F(5)	8*d*	1	1	0.2860(8)	0.4052(6)	0.5583(7)	0.0464(16)
F(6)	8*d*	1	1	0.0057(7)	0.5954(6)	0.4893(7)	0.0415(14)
F(7)	4*c*	*.m.*	1	0.1558(12)	3/4	0.3077(12)	0.056(3)
F(8)	4*c*	*.m.*	0.36(4)	-0.192(4)	3/4	0.600(4)	0.059(12)
H(1)	4*c*	*.m.*	1	0.06(2)	3/4	0.41(2)	0.05

Table S25: Anisotropic displacement parameters U^{ij} of $Ba[U_2F_{12}]\cdot HF$ at 100 K.

Atom	U^{11} / Å²	U^{22} / Å²	U^{33} / Å²	U^{23} / Å²	U^{13} / Å²	U^{12} / Å²
Ba(1)	0.0397(5)	0.0368(4)	0.0364(4)	0	0.0033(4)	0
U(1)	0.0340(2)	0.0404(2)	0.0335(2)	0.00122(17)	-0.00037(16)	0.00011(16)
F(1)	0.041(3)	0.042(4)	0.043(4)	-0.005(3)	-0.006(3)	-0.005(3)
F(2)	0.038(3)	0.046(4)	0.039(3)	0.006(3)	0.001(3)	0.000(3)
F(3)	0.038(3)	0.042(4)	0.040(3)	-0.001(3)	-0.001(3)	0.004(3)
F(4)	0.040(3)	0.051(4)	0.039(3)	0.006(3)	0.000(3)	-0.008(3)
F(5)	0.042(3)	0.060(4)	0.037(3)	-0.008(3)	-0.003(3)	0.012(3)
F(6)	0.044(3)	0.042(3)	0.039(3)	-0.001(3)	-0.004(3)	0.000(3)
F(7)	0.058(7)	0.032(16)	0.039(5)	0	-0.002(5)	0

F(8)	0.06(2)	0.032(16)	0.08(3)	0	-0.002(18)	0

Powder X-ray diffraction patterns of the hexafluoridouranates(V)

$LiUF_6$

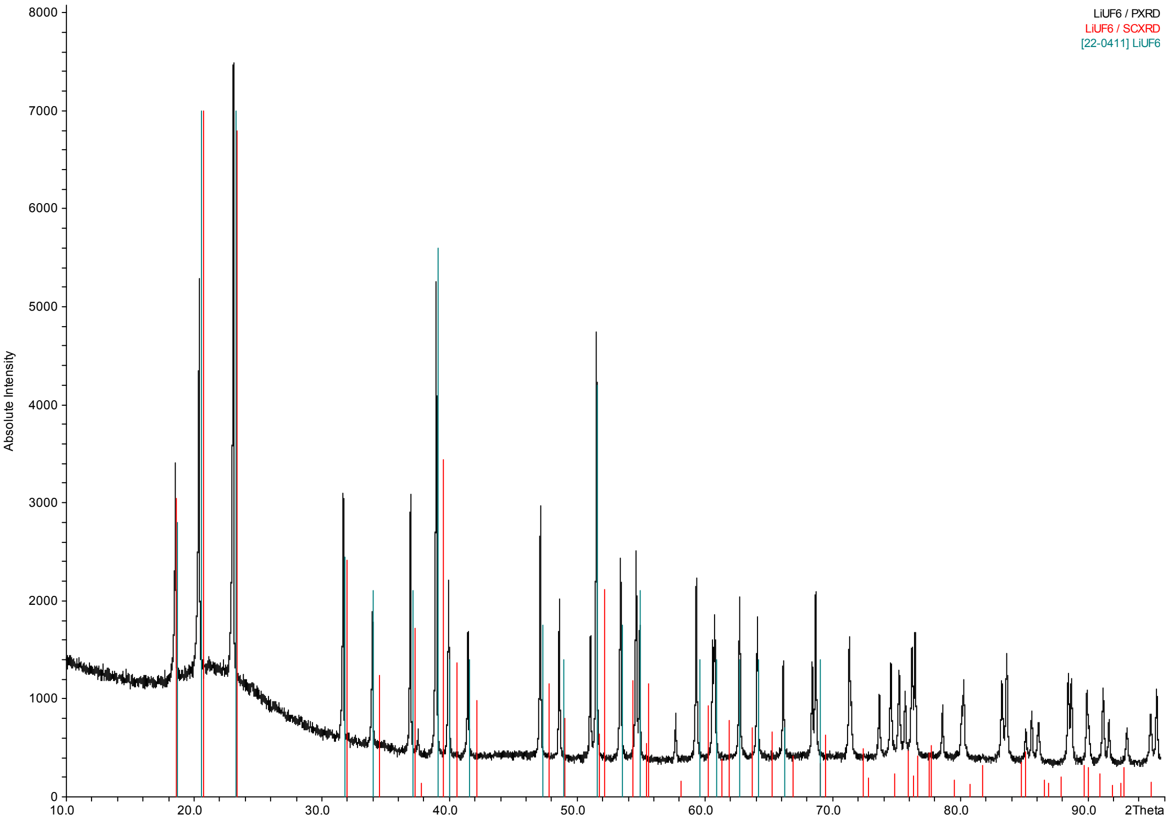

Figure S4: Powder X-ray diffractogram of $LiUF_6$ (black). Calculated reflex positions and intensities in red based on the single crystal structure of $LiUF_6$. ICDD entry [22-0411] of $LiUF_6$ in green.[1] Measurement in capillary with 0.3 mm diameter, 0.25° increment 2θ and 26 s exposure time.

$NaUF_6$ – rhombic and cubic polymorphs

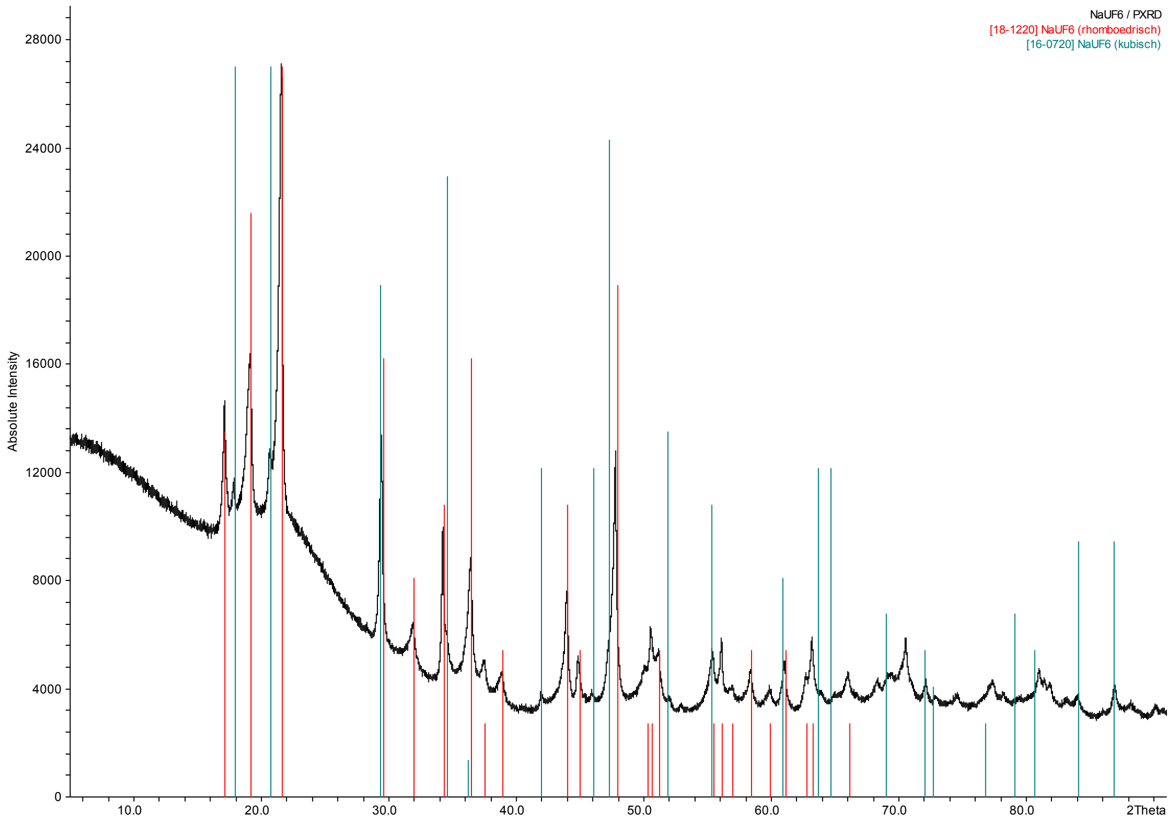

Figure S5: Powder X-ray diffractogram of $NaUF_6$ (black). Reflex positions and intensities of ICDD entry [16-0720] of $NaUF_6$ (cubic polymorph) in red, of ICDD entry [18-1220] of $NaUF_6$ (rhombic polymorph) in green.[1,2] Measurement in capillary with 0.3 mm diameter, 0.1° increment 2θ and 68 s exposure time.

KUF_6

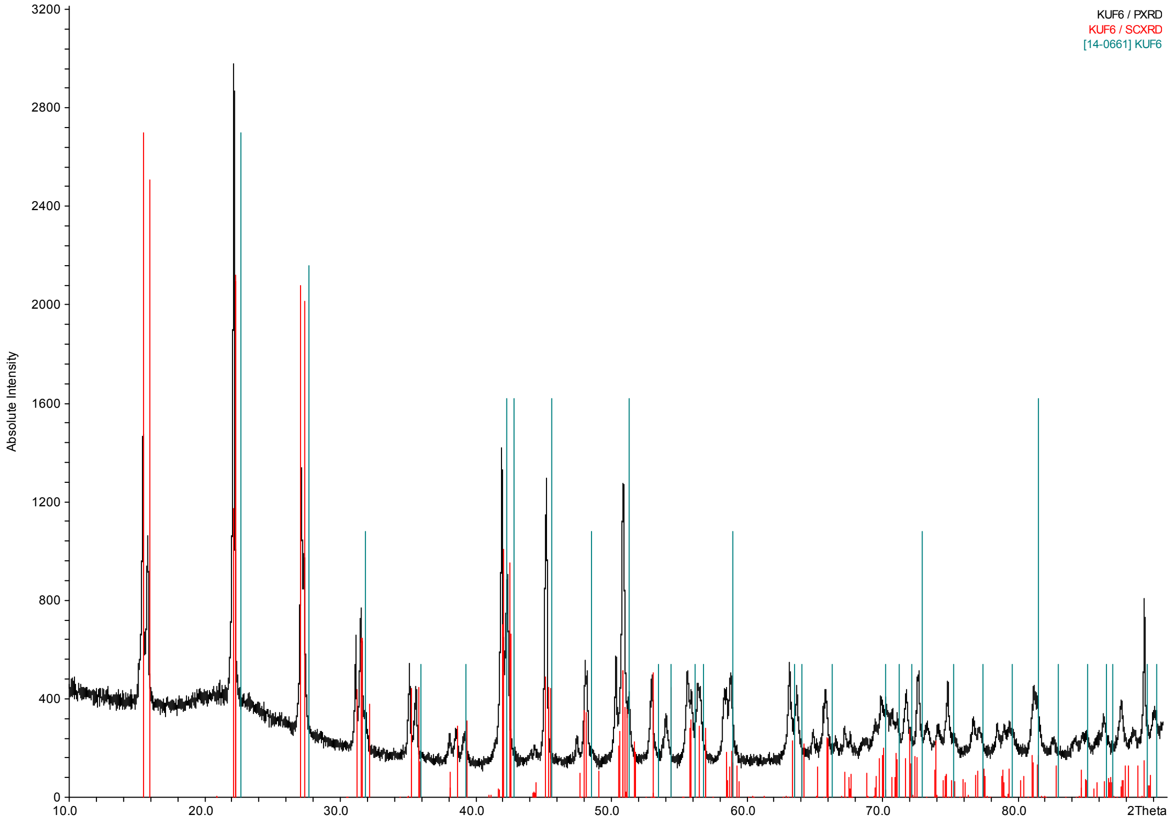

Figure S6: Powder X-ray diffractogram of KUF_6 (black). Calculated reflex positions and intensities based on our single crystal structure of KUF_6 in red, of ICDD entry [14-0661] of KUF_6, the previous model, in green.[3] Measurement in capillary with 0.3 mm diameter, 0.5° increment 2θ and 33 s exposure time.

$RbUF_6$

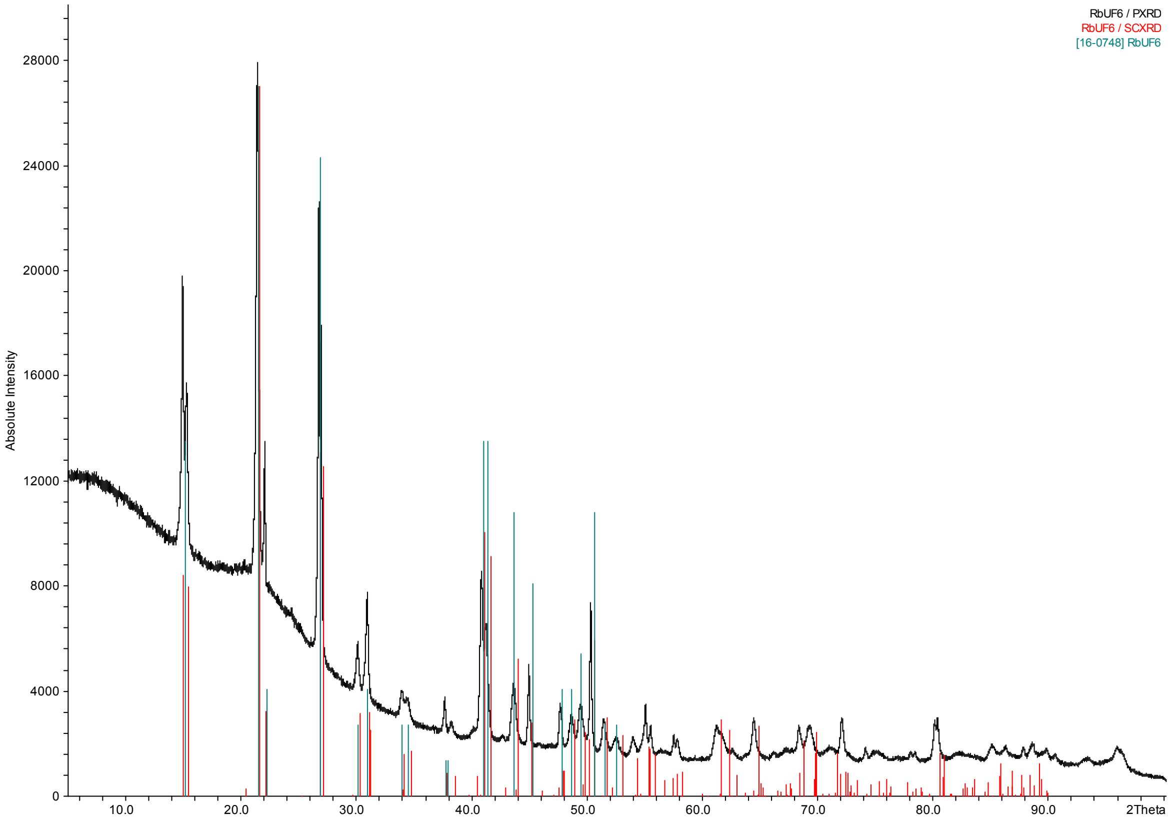

Figure S7: Powder X-ray diffractogram of $RbUF_6$ (black). Calculated reflex positions and intensities based on our single crystal structure of $RbUF_6$ in red, of ICDD entry [16-0748] of $RbUF_6$ in green.[1] Measurement in capillary with 0.3 mm diameter, 0.1° increment 2θ and 47 s exposure time.

$CsUF_6$

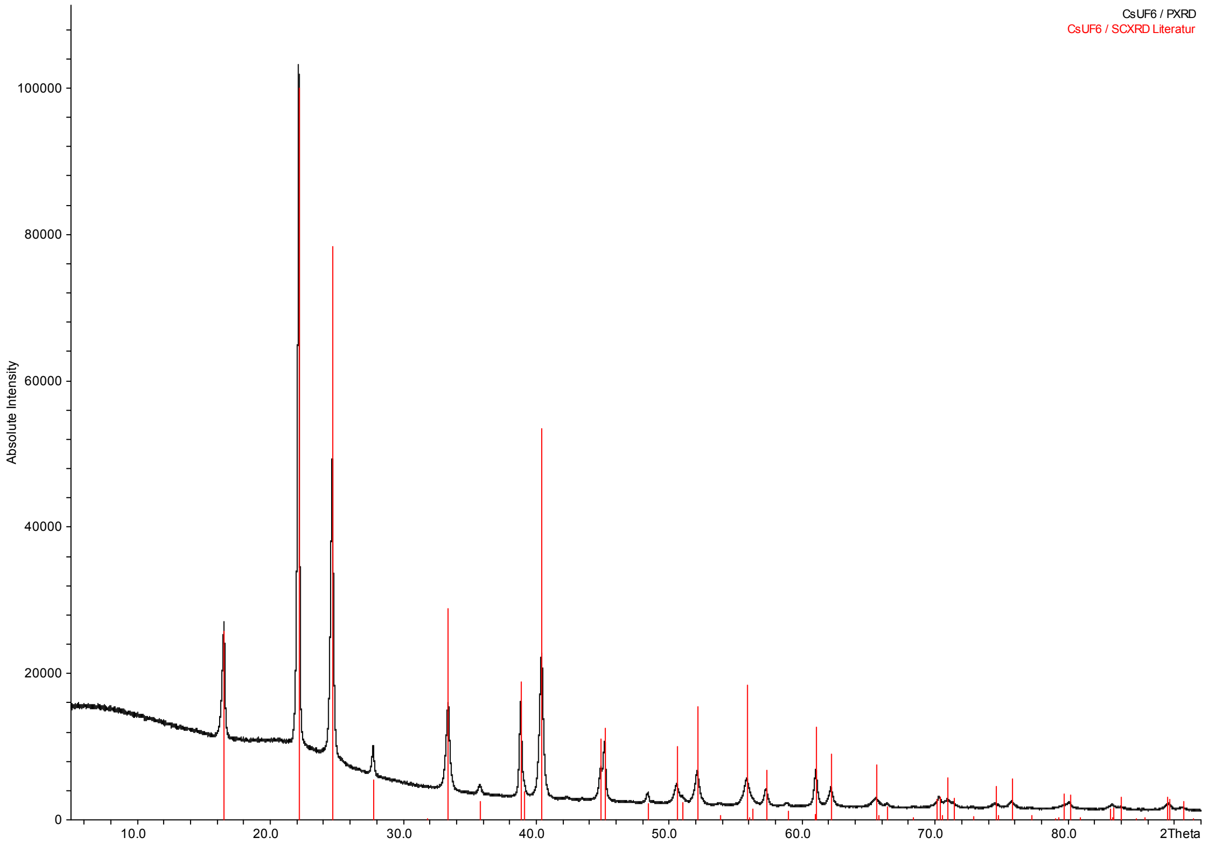

Figure S8: Powder X-ray diffractogram of $CsUF_6$ (black). Calculated reflex positions and intensities based on the single crystal structure of $CsUF_6$ in red.[4] Measurement in capillary with 0.3 mm diameter, 0.1° increment 2θ and 37 s exposure time.

$TlUF_6$

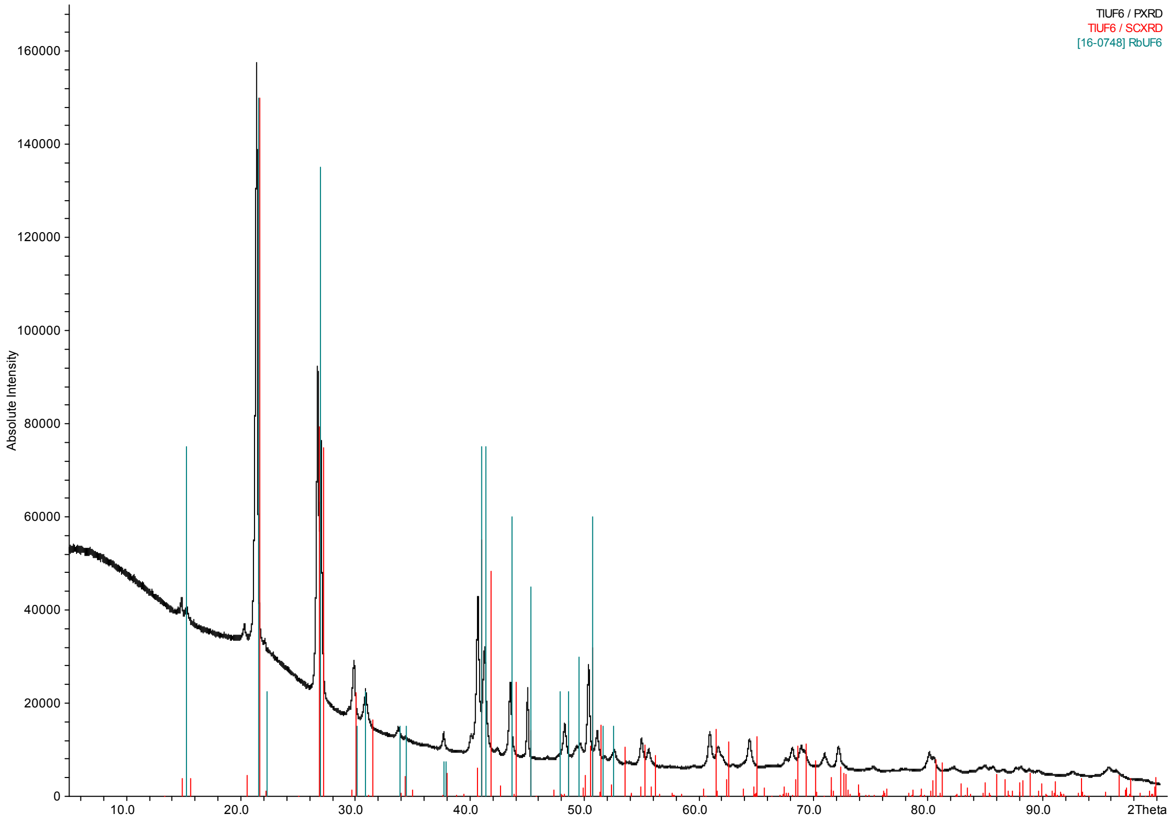

Figure S9: Powder X-ray diffractogram of $TlUF_6$ (black). Calculated reflex positions and intensities based on our single crystal structure of $TlUF_6$ in red, of the ICDD entry [16-0748] of $RbUF_6$ (isotypic) in green.[1] Measurement in capillary with 0.3 mm diameter, 0.1° increment 2θ and 45 s exposure time.

H_3OUF_6

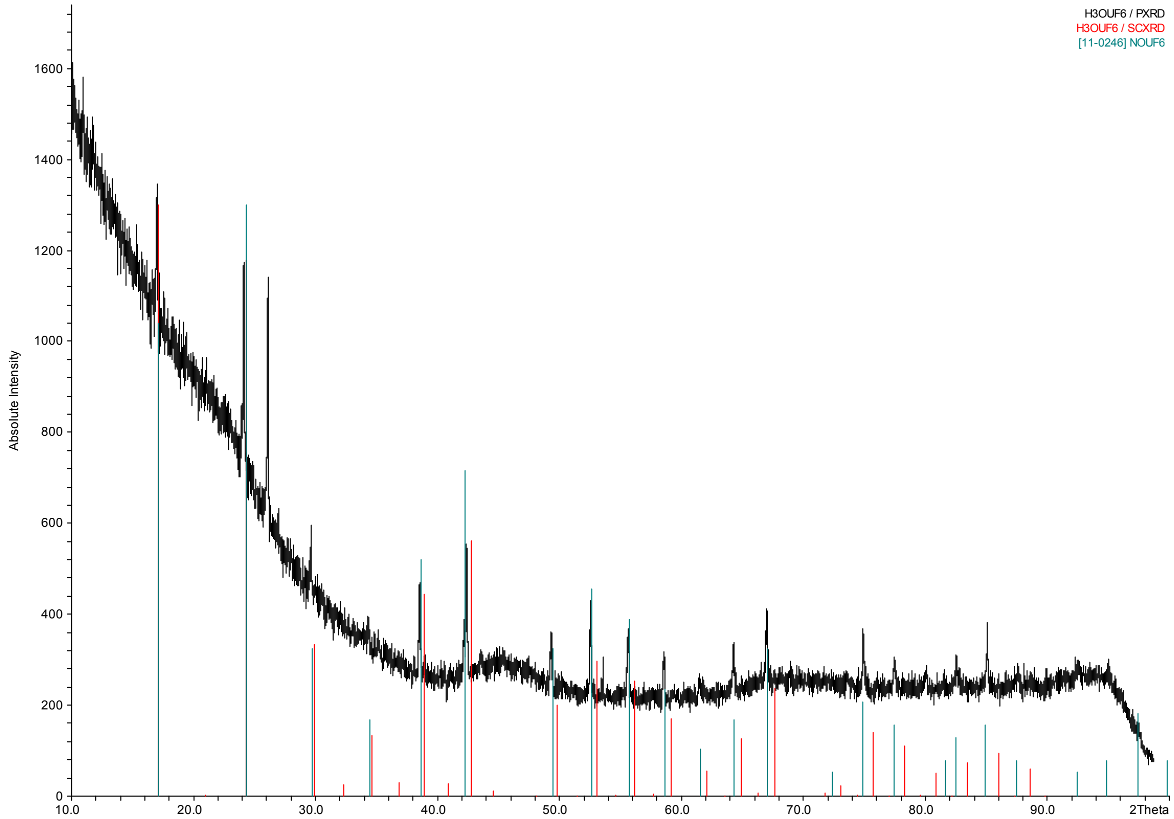

Figure S10: Powder X-ray diffractogram of H_3OUF_6 (black). Calculated reflex positions and intensities based on our single crystal structure of H_3OUF_6 in red, of the ICDD entry [11-0246] of $NOUF_6$ in green.[5] Measurement in capillary with 0.3 mm diameter, 0.4° increment 2θ and 40 s exposure time.

Characterization of UF_5 used as a starting material

Using the ICDD database, the reflexes in the diffractogram can be assigned to those reported for β-UF_5 [72-0959], which crystallizes in the tetragonal crystal system in the space group $I\bar{4}2d$ (No. 122), see Figure S11.[6] A neutron diffraction study resulted in the lattice parameters a = 11.473(3), c = 5.208(2) Å, V = 685.53 Å^3 (T = 293 K).[6] Indexing the reflections led to the lattice parameters a = 11.4763(9), c = 5.2040(5) Å, V = 685.39(1) Å^3. These agree with those determined by neutron diffraction.

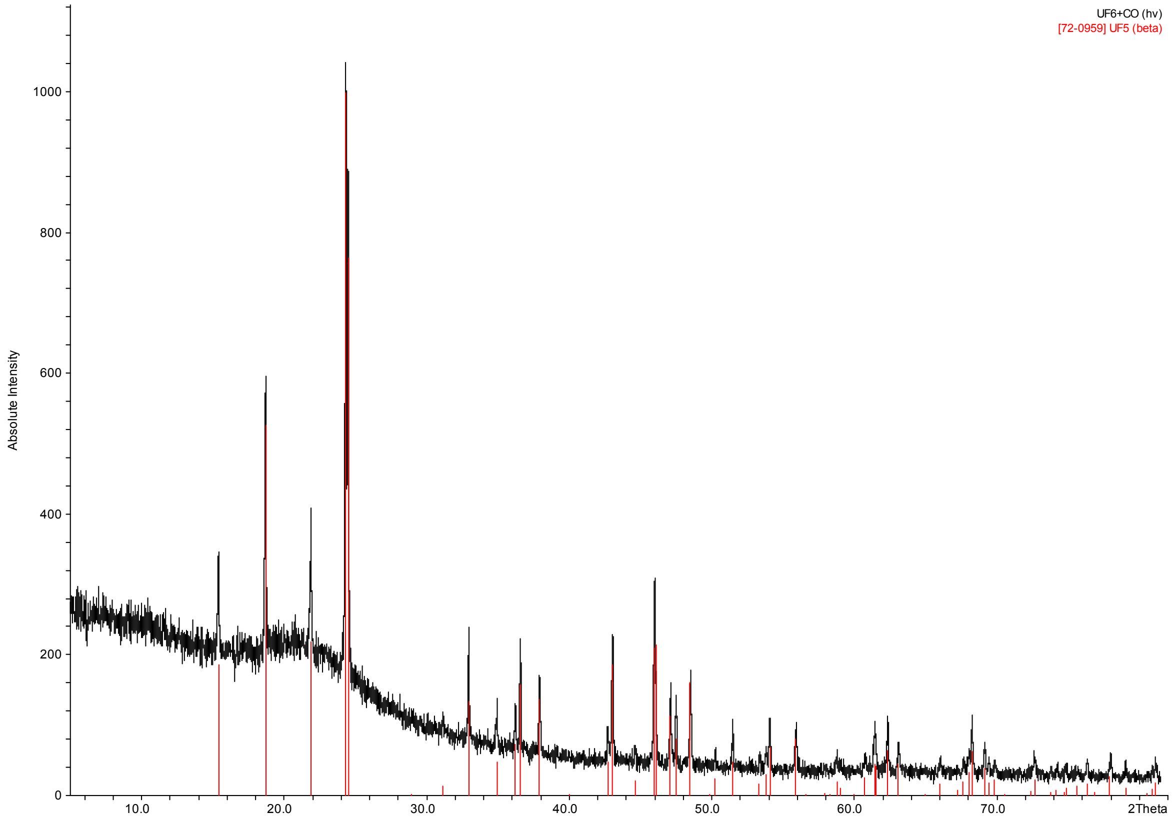

Figure S11: Powder X-ray diffraction pattern of β–UF_5 (black) obtained by reaction of UF_6 and CO under UV light irradiation. ICDD entry [72-0959] of β–UF_5 with calculated reflex positions and intensities in red for comparison.[6] Measurement in capillary with 0.3 mm diameter, 1.5° step size 2θ and 8 s exposure time.

The IR spectrum of the obtained *β*–uranium(V) fluoride is shown in Figure S12. The band positions are given in Table S26. The IR spectrum shows two strong bands, the most intensive one is at 569 cm^{-1} with a shoulder at 608 cm^{-1}. These agree with reported band positions at 573 and 605 cm^{-1}.[7] The shoulder band was assigned to a common mode vibration of the terminally bound F atoms that is also Raman active. The most intensive band should be due to the asymmetric stretch vibration ν_{as}(U–F). The medium strong band at 512 cm^{-1} is likely the symmetric stretch vibration ν_s(U–F).[7]

Table S26: Absorption bands of the ATR-FTIR spectrum of *β*–UF_5 recorded at r.t. with band assignments.

Wave number / cm^{-1}	Assignment
608	ν (U–F)
569	ν_{as}(U–F)
512	ν_s(U–F)

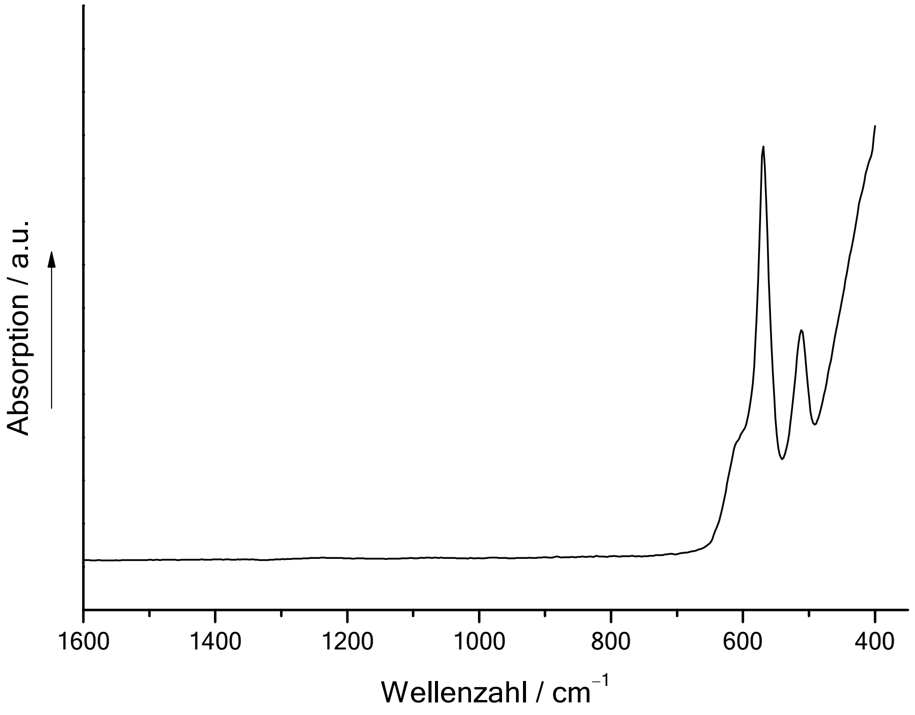

Figure S12: ATR-IR spectrum of *β*-UF_5 recorded at room temperature with 32 scans and a resolution of 4 cm^{-1}.

Literature

[1] R. A. Penneman, G. D. Sturgeon, L. B. Asprey, *Inorg. Chem.* **1964**, *3*, 126–129.
[2] G. D. Sturgeon, R. A. Penneman, F. H. Kruse, L. B. Asprey, *Inorg. Chem.* **1965**, *4*, 748–750.
[3] L. B. Asprey, F. H. Kruse, A. Rosenzweig, R. A. Penneman, *Inorganic Chemistry* **1966**, *5*, 659–661.
[4] A. Rosenzweig, D. T. Cromer, *Acta Crystallogr.* **1967**, *23*, 865–867.
[5] F. J. Musil, P. R. Ogle, K. E. Beu, *Powder X-Ray Diffraction and Related Data on NOUF6 and NOMoF6, GAT-T-553*, Goodyear Atomic Corp., **1958**.
[6] J. C. Taylor, A. B. Waugh, *J. Solid State Chem.* **1980**, *35*, 137–140.
[7] E. Jacob, *Z. Anorg. Allg. Chem.* **1973**, *400*, 45–50.
